# Long-range spectral entanglement and quantum correlations in highly nondegenerate multi-comb and ultrafast systems

**DOI:** 10.1038/s41377-026-02491-3

**Published:** 2026-11-05

**Authors:** Sahil Pontula, Debasmita Banerjee, Marin Soljačić, Yannick Salamin

**Affiliations:** 1 Department of Physics, MIT, Cambridge, MA 02139 USA; 2 Department of Electrical Engineering and Computer Science, MIT, Cambridge, MA 02139 USA; 3 Research Laboratory of Electronics, MIT, Cambridge, MA 02139 USA; 4 CREOL, The College of Optics and Photonics, University of Central Florida, Orlando, FL 32816 USA

**Keywords:** Frequency combs, Quantum optics

## Abstract

Frequency combs are multimode photonic systems that underlie countless precision sensing and metrology applications. Since their invention over two decades ago, numerous efforts have pushed frequency combs to broader bandwidths and more stable operation. More recently, quantum squeezing and entanglement have been explored in single-comb systems for continuous-variable quantum computing and quantum-enhanced spectroscopy. However, the production of bright quantum light across multiple nondegenerate pulses or frequency combs, a key technology for multispectral applications, remains largely unexplored. In this work, we explore a mechanism that generates a series of nonlinearly coupled bright frequency combs through cascaded three-wave upconversion and downconversion processes mediated by a single idler comb. We consider both discrete combs and broadband ultrafast pulse systems, showing how these systems generate inter- and intracomb two-mode squeezing and entanglement directly in the frequency basis, spanning a large spectral range. Furthermore, we demonstrate how these systems can be engineered to produce target cluster states and concentrated two-mode squeezing through covariance matrix optimization. Our findings uncover a new multimode quantum regime enabled by hierarchical nonlinear interactions in two frequency dimensions, unlocking applications such as entanglement-assisted spectroscopy, squeezing-enhanced pump-probe measurements, quantum spectral multiplexing, and spectrally addressable cluster state generation for quantum information science.

## Abstract (web summary)

A cavity with idler-mediated cascaded second-order nonlinearity generates a nondegenerate system of nested frequency combs, enabling long-range quantum squeezing across distinct spectral bands and multipartite entanglement for cluster states.

## Graphical abstract

**Figure:**
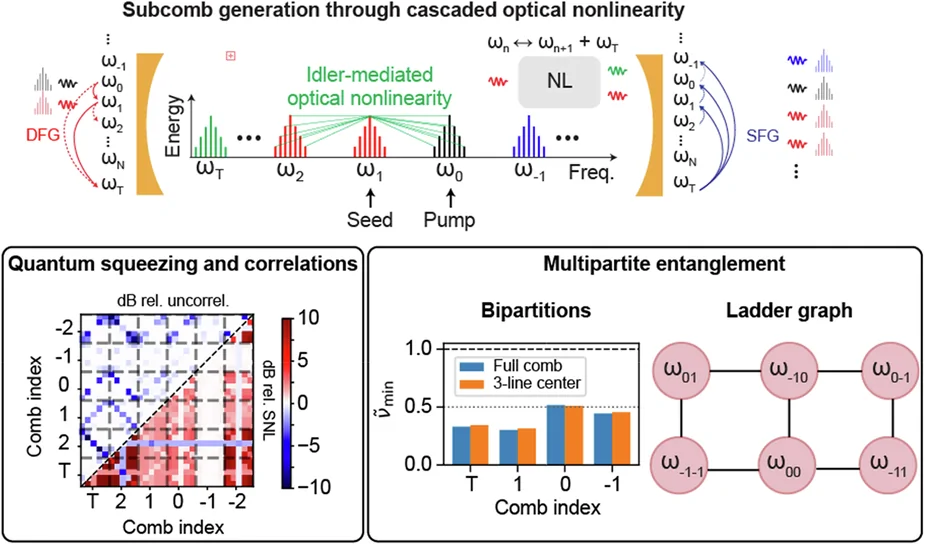

## Introduction

Optical frequency combs have revolutionized precision metrology, providing a coherent link between the optical and microwave domains while enabling advances in spectroscopy, timing, and sensing^1,2^. In parallel, nonlinear optical interactions have become the subject of growing interest for generating quantum light in multimode optical systems^3,4^. For example, strong quantum correlations in multimode systems can manifest as noise reduction below the standard quantum limit or shot noise limit (SNL) in appropriate “supermodes” and quadrature combinations^5,6^, offering pathways for quantum-enhanced sensing protocols. Consequently, continuous-variable (CV) entanglement and squeezing in frequency comb systems have attracted significant attention due to their potential for quantum-enhanced measurements and quantum information processing^6–9^. Quantum comb line correlations and CV multipartite entanglement have been demonstrated in both bulk (e.g., synchronously pumped optical parametric oscillators)^6,10–12^ and integrated (e.g., microresonator) systems^4,13–21^. Furthermore, multimode entanglement and cluster states in frequency combs and optical parametric oscillators^6,12,13,22–26^ show rapid progress towards large, scalable frequency-multiplexed quantum resources for computation, networks, and metrology. Beyond single combs, two-comb systems have found key applications in dual comb spectroscopy and have recently been extended to the quantum domain^7–9,27,28^. However, quantum correlations and entanglement in all of these systems remain restricted to a single comb or to a limited spectral range. In addition, the majority of work has focused on squeezed supermode generation in below-threshold systems.

To address this gap, we investigate the quantum optics of bright multi-comb systems by examining CV entanglement and quantum correlations in a nonlinear system that generates a comb of bright frequency combs—a “comb of combs.” Such nested comb structures, with two frequency dimensions, introduce additional degrees of freedom and coupling pathways that fundamentally alter the multimode quantum landscape compared to single comb systems. We demonstrate that the nonlinear processes that couple different combs create long-range entanglement and quantum correlations that span both the intra-comb and inter-comb domains. Intriguingly, these quantum behaviors extend to modes that do not couple directly via the nonlinear mechanism, showcasing the important role of the shared idler comb that mediates nonlinear interactions across the rest of the optical combs. These quantum correlations manifest as complex squeezing patterns in collective quadratures that encompass modes from multiple combs. We emphasize that, in contrast to the substantial effort devoted to generating single ultra-broadband frequency combs^29–35^, our focus is instead on the quantum optics of multiple distinct but coupled frequency combs. This multi-comb architecture is qualitatively different from a single broadband comb: rather than concentrating bandwidth into one comb, it distributes correlations across several interacting combs that can collectively span a large spectral range, opening the door to quantum-enhanced multispectral applications.

The physical underpinnings of our multi-comb system lie in cascaded three-wave mixing (TWM) processes. The TWMs are mediated by a common low-frequency idler comb (initially generated by difference frequency generation between two optical combs) that propagates in the system and distributes energy into new upconverted and downconverted combs. In addition to exploring entangled frequency comb generation, we show how our proposed physical mechanism addresses an outstanding challenge in quantum frequency combs: generating bright quantum light directly in the frequency-mode basis, which is most relevant for spectroscopy applications. To complement our discrete coupled-mode analysis, we move beyond discrete frequency-multimode systems and study the quantum optics of ultrafast nonlinear systems harboring multiple broadband nondegenerate pulses. The interpulse correlations we uncover in this ultrafast regime may form a promising resource for quantum-enhanced pump-probe measurements. Finally, we showcase the potential of pulse shaping and dissipation engineering (i.e., using loss as a resource) to tailor the multimode covariance matrix for objectives such as achieving strong squeezing between a particular pair of wavelengths.

The quantum structure of this multi-comb, multi-pulse architecture opens several routes toward quantum-enhanced applications. In particular, the hierarchical entanglement studied here could provide a basis for entanglement-assisted spectroscopy with multiple bright combs and for spectrally multiplexed CV quantum information protocols. More broadly, because many practical comb platforms operate in bright, above-threshold regimes rather than as weak squeezed-vacuum sources, our results identify a route to generating multimode states with strong quantum correlations in operating regimes relevant for low-noise sensing and signal processing. These findings provide a framework for analyzing and exploiting quantum correlations in coupled multi-comb systems.

## Results

### Cascaded comb generation in a multimode nonlinear cavity

**Fig. 1:**
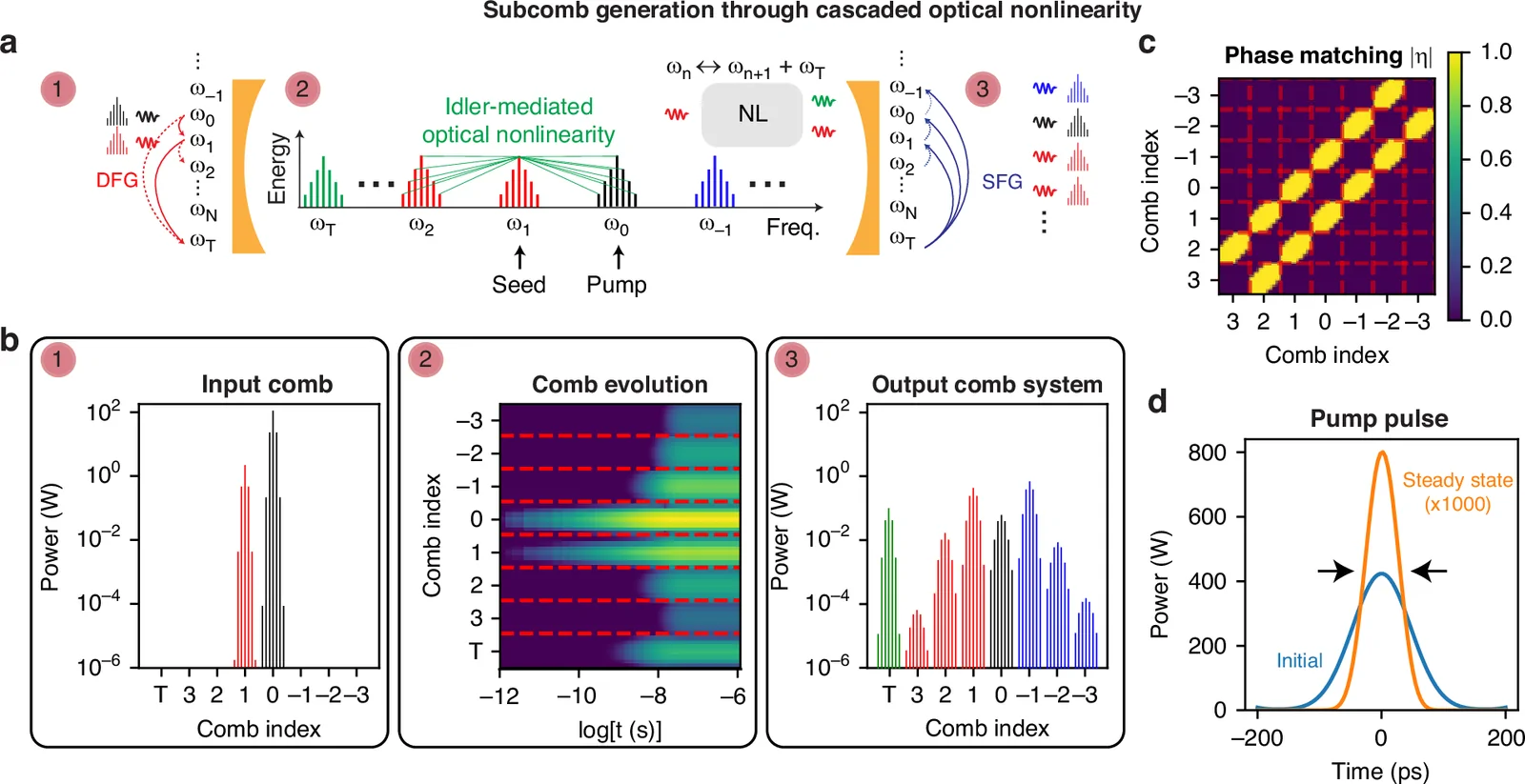
Cascaded comb generation in a multimode cavity. **a** A multimode system is pumped with a comb centered at frequency *ω*_0_ and seeded with either a single frequency or comb centered at frequency *ω*_1_. DFG between the pump and seed generates an idler comb at frequency *ω*_*T*_, which is then recycled to generate “subcombs” through cascaded sum and difference frequency generation (SFG/DFG) processes. Taken together, the subcombs comprise a “primary comb” with frequency spacing *ω*_*T*_. This system can be realized in a multimode cavity that is synchronously pumped/seeded by a mode-locked or modulated continuous wave laser, which gives rise to the subcomb frequency spacing *ω*_*m*_ ≪ *ω*_*T*_, where *ω*_*m*_ = *nω*_FSR_ coincides with a multiple of the cavity free spectral range. Cascaded nonlinear processes produce the primary comb with spacing *ω*_*T*_ centered around the pump subcomb at frequency *ω*_0_. **b** Cascaded comb generation in a multimode cavity with *I* = 7 subcombs, each with *J* = 7 modes. **c** Heatmap of phase matching factor between individual modes, including dispersion and assuming multi-poled lithium niobate (see SI)^53^. **d** Narrowing of the pump pulse due to broadening of its frequency comb from the cascaded three-wave mixing mediated by the idler comb. All optical comb modes have outcoupling loss 5% and intrinsic loss 5%, the idler comb has outcoupling loss 10% and intrinsic loss 10%, and *β*_0_ = 10^−4^ J^−1/2^. The pump wavelength is *λ*_0_ = 465 nm, the idler wavelength is *λ*_*T*_ = 4.07 *μ*m (*ω*_*T*_ = 2*π* ⋅ 73.7 THz), and the intracomb spacing is *ω*_*m*_ = 2*π* ⋅ 3.00 GHz (coinciding with the FSR of a 10 cm free space cavity). The optical subcombs lie at wavelengths 708, 603, 525, 465, 417, 378, and 346 nm. In (**b**), “T” denotes the idler comb

The physical mechanism we explore is depicted in Fig. 1a, which features cascaded comb generation in a multimode nonlinear cavity. In the most general case, a pump and seed comb centered at frequencies *ω*_0_ ≡ *ω*_00_ and *ω*_1_ ≡ *ω*_10_ are injected into the cavity and undergo TWM to generate an idler comb centered at *ω*_*T*_ = *ω*_0_ − *ω*_1_. The idler comb is then recycled in the cavity to generate blueshifted (*ω*_*n*<0_) and redshifted (*ω*_*n*>0_) combs relative to *ω*_0_ through nonlinear upconversion and downconversion processes^36,37^.

Figure 1 features the cascaded generation of “subcombs” through sum/difference frequency generation processes (SFG/DFG). The subcombs collectively comprise a “primary comb” which has spacing *ω*_*T*_ ≡ *ω*_*T*0_ equal to the center frequency of the common idler comb that mediates the cascaded nonlinear processes. The system is pumped with a comb centered at frequency *ω*_0_ with comb line spacing *ω*_*m*_ ≪ *ω*_*T*_ (which can come from synchronized pumping of a multimode cavity by a mode-locked or electro-optically modulated laser). We note that the condition *ω*_*m*_ ≪ *ω*_*T*_ can be satisfied for a wide variety of systems across the electromagnetic spectrum. Unless otherwise specified, we will consider in the main text *ω*_*T*_ to lie in the mid-infrared regime, allowing the generation of combs spanning the UV to IR range where phase matching is achievable using commercial *χ*^(2)^ crystals. We will consider periodically poled lithium niobate here, the relevant properties of which are detailed in the SI.

Because the idler comb couples all of the *ω*_*m*_-spaced subcombs, one may anticipate nontrivial correlations and entanglement to emerge between discrete modes of different subcombs. Writing the Hamiltonian for this nonlinear multimode system in the discrete frequency mode basis (see Methods for details), we obtain the following Heisenberg equations of motion in the mean field that describe the system dynamics:

1 $${\dot{a}}_{-k}^{T} =	\sum\limits_{ij}{\beta }_{ijk}{a}_{ij}^{* }{a}_{i-1,j-k}-({\gamma }_{T}+{\mu }_{T}){a}_{-k}^{T}\\ {\dot{a}}_{ij} =	\sum\limits_{k}\left[-{\beta }_{i+1,j+k,k}^{* }{a}_{-k}^{T}{a}_{i+1,j+k}+{\beta }_{ijk}{a}_{-k}^{T* }{a}_{i-1,j-k}\right]\\ 	-({\gamma }_{ij}+{\mu }_{ij}){a}_{ij}+\sqrt{2{\gamma }_{ij}}{s}_{ij}$$

Here, ${a}_{-k}^{T}$ denotes the mode in the idler comb at frequency ${\omega }_{-k}^{T}={\omega }_{T0}+k{\omega }_{m}$ and *a*_*ij*_ indexes mode *j* in subcomb *i* with frequency *ω*_*ij*_ = *ω*_00_ − *iω*_*T*0_ − *jω*_*m*_ (the notation is such that *ω*_00_ → *ω*_*ij*_ corresponds to the *creation* of idler photons whose total energy is *iℏω*_*T*0_ + *jℏω*_*m*_). As described in the SI, ${\beta }_{ijk}={\beta }_{0}{\eta }_{ijk}\sqrt{\hslash {\omega }_{ij}{\omega }_{i-1,j-k}{\omega }_{-k}^{T}}$ denotes the nonlinear strength, with *β*_0_ related to the nonlinear susceptibility *χ*^(2)^ of the cavity’s nonlinear crystal that supports the cascaded SFG/DFG processes. The phase matching prefactor ${\eta }_{ijk}={e}^{i\Delta {K}_{ijk}{L}_{{{{\rm{cry}}}}}/2}{{{\rm{sinc}}}}(\Delta {K}_{ijk}{L}_{{{{\rm{cry}}}}}/2)$, where $\Delta {K}_{ijk}={K}_{i-1,j-k}-{K}_{ij}-{K}_{-k}^{T}-{K}_{{{{\rm{link}}}}}^{i}$ is the wavevector mismatch (${K}_{{{{\rm{link}}}}}^{i}$ is the relevant crystal grating vector from the poling design) and *L*_cry_ is the crystal length, governs the strength of nonlinear coupling between individual frequency modes. *γ*_*ij*_, *μ*_*ij*_ respectively denote outcoupling and intrinsic loss for each comb mode (while *γ*_*T*_, *μ*_*T*_ denote the corresponding losses for the idler comb, assumed constant across the comb for simplicity) and *s*_*ij*_ denotes the input drive amplitude at *ω*_*ij*_. Unless otherwise specified, *s*_*i*∉{0, 1},*j*_ = 0 and *γ*_*ij*_ → *γ*_*i*_, *μ*_*ij*_ → *μ*_*i*_ are independent of the intracomb index (which is reasonable given that the individual combs we consider here have relatively small fractional bandwidth).

In Fig. 1b, we explore how the multimode nonlinear system takes the energy in the initial pump comb and uses it to create combs at different frequencies, both blueshifted and redshifted relative to the pump. Simultaneously, three-wave mixing interactions between adjacent combs populate the idler comb (green). The strength of mixing between individual frequency modes is governed by phase matching, as shown in Fig. 1c. This phase matching heatmap determines which modes mix within the phase matching bandwidth and thereby sets the extent and shape of the idler comb and other subcombs (for smaller *ω*_*m*_ or shorter crystals (larger phase matching bandwidth), more modes between adjacent combs are allowed to mix with one another). Changing *ω*_*m*_ modifies the effective phase mismatch sampled by the comb lines, and thus acts as a simple form of dispersion engineering that can significantly impact both the mean field and quantum noise behavior of the system, as we explore in the SI. We explicitly consider a phase matching scheme that phase matches DFG between the center comb lines of nearest-neighbor optical combs, achievable with a nonlinear crystal with multiple poling periods as described in the SI.

A side effect of the cascaded comb generation is the spectral broadening of each comb, as subsequent three-wave mixing processes populate comb lines further from the center. This is shown in Fig. 1d for the pump, where this spectral broadening manifests as temporal narrowing of the pulse profile. It is in this strong cascading regime that we expect to see signatures of strong quantum correlations and entanglement.

### Two-mode quantum correlations and squeezing

By linearizing the full Heisenberg-Langevin equations of motion about the steady state obtained by solving Eq. (1) (see Methods), we calculate the intracavity fluctuations $\delta {\hat{a}}_{k},\delta {\hat{a}}_{k}^{{{\dagger}} }$ for each mode. The output fluctuation (annihilation) field operators $\delta {\hat{s}}_{k,{{{\rm{out}}}}}=\sqrt{2{\gamma }_{k}}\delta {\hat{a}}_{k}-\delta {\hat{s}}_{k,{{{\rm{in}}}}}$ can then be computed from the input-output relation of temporal coupled mode theory^38^. Output photon flux and intensity fluctuations can be derived from the fluctuations of ${\hat{N}}_{k}={\hat{s}}_{k,{{{\rm{out}}}}}^{{{\dagger}} }{\hat{s}}_{k,{{{\rm{out}}}}}$ and ${\hat{P}}_{k}=\hslash {\omega }_{k}{\hat{N}}_{k}$, respectively. In all examples, noise observables will be computed at 1 MHz (which lies well within the flat noise frequency region where the broadband correlations take effect).

**Fig. 2:**
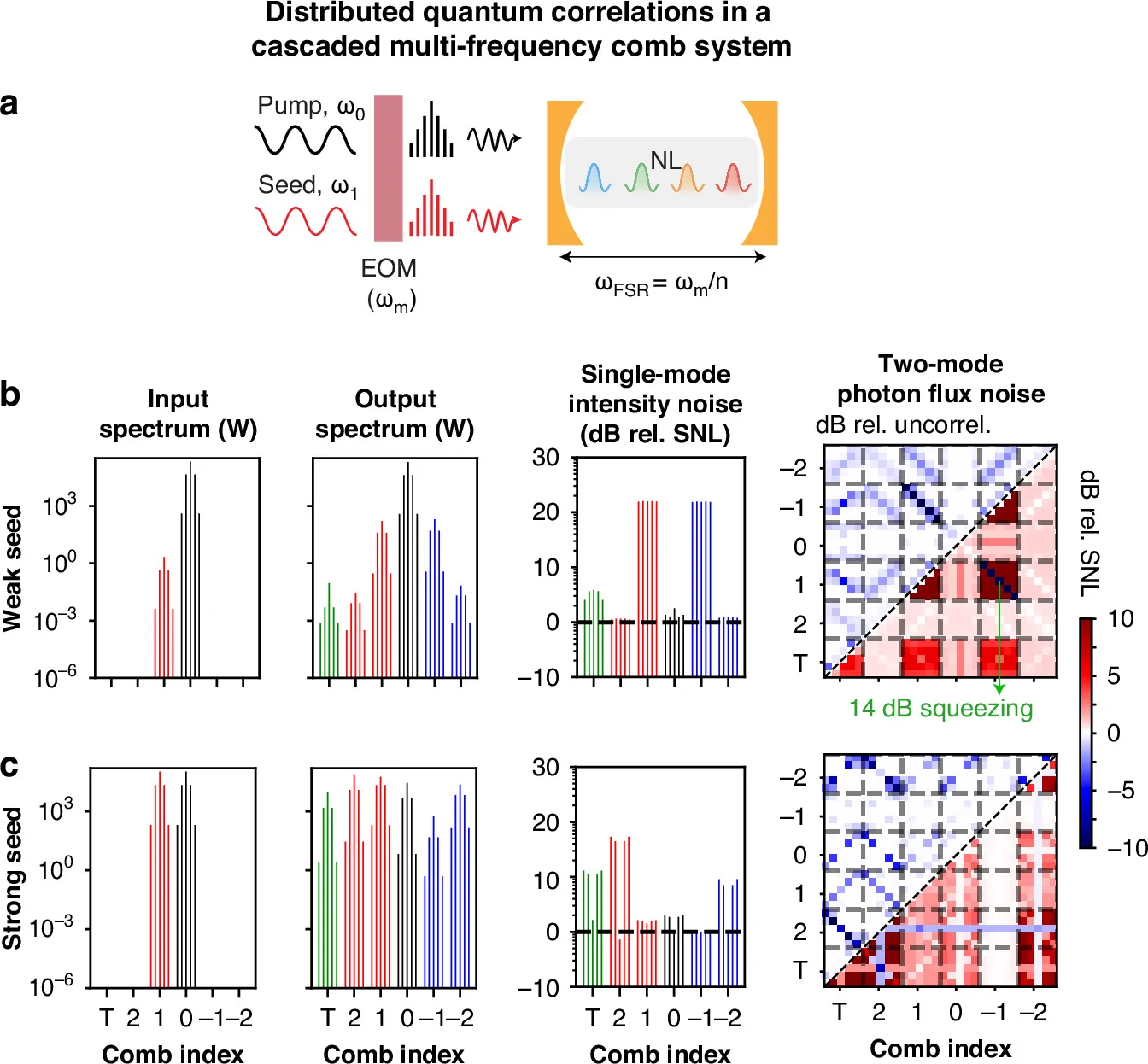
Quantum correlations and multimode entanglement in a multi-comb system generated by cascaded second-order nonlinear processes. **a** Schematic illustration of cascaded comb generation in a nonlinear (NL) multimode cavity pumped by an electro-optic modulated (EOM) quasi-continuous wave source, whose modulation frequency coincides with a multiple of the cavity free spectral range *ω*_*m*_ = *nω*_FSR_. **b** Input and output spectra, single-mode intensity noise (dB relative to shot noise limit), and two-mode photon flux noise (dB relative to uncorrelated and shot noise limits) for a strongly pumped and weakly seeded system. **c** Corresponding plots for a strongly pumped and strongly seeded system. In this system, *I* = *J* = 5, optical modes have 1% intrinsic loss and 50% outcoupling roundtrip loss ((*γ* + *μ*)/*ω*_FSR_ ≈ 0.08 so coupled mode theory is still valid), while the idler modes have 1% intrinsic loss and 2.5% outcoupling roundtrip loss. Large outcoupling relative to intrinsic loss is chosen so that the escape efficiency *γ*/(*γ* + *μ*) approaches unity, which is what limits observable squeezing

In Fig. 2, we explore the mean field and quantum noise behavior of a system that in steady state possesses *I* = 5 subcombs, each with *J* = 5 comb lines. Figure 2a presents a schematic illustration of one possible platform for this system, where an electro-optic modulated quasi-continuous wave source pumps a nonlinear multimode cavity. The modulation rate sets the spacing *ω*_*m*_ of modes within each subcomb. The modulator can also be placed inside the cavity to increase the subcomb bandwidth^39^. Note that *ω*_*m*_ should be chosen to correspond to a multiple of the cavity free spectral range, *ω*_*m*_ = *nω*_FSR_, so that all comb modes are resonant in the cavity. The interplay between intracavity modulation (which introduces linear coupling between the modes in individual subcombs) and the optical nonlinearity (which couples subcombs) may enable topological states of quantum light^37^. Finally, the intracavity nonlinear crystal is phase matched for cascaded SFG/DFG (see SI for system design).

Figure 2b, c shows the mean-field spectra as well as single- and two-mode noise for the cases of “weak” (relative to the pump) and “strong” seeds, respectively. Note that most modes are individually above the shot noise limit because of the simultaneous noise coupling between modes from SFG and DFG processes, though a stronger seed tends to quench the single-mode intensity noise. The two-mode photon flux noise plots show the normalized gain-balanced photon flux difference, which can also be interpreted as the noise reduction factor

2 $${{{{\rm{NRF}}}}}_{mn}=\frac{{{{\rm{Var}}}}({\hat{N}}_{m}-{g}_{mn}{\hat{N}}_{n})}{{{{\rm{norm.}}}}}$$

where ${{{\rm{norm.}}}}=\langle {\hat{N}}_{m}\rangle +{g}_{mn}^{2}\langle {\hat{N}}_{n}\rangle$ for a shot noise (and uncorrelated) normalization and ${{{\rm{norm.}}}}={{{\rm{Var}}}}({\hat{N}}_{m})+{g}_{mn}^{2}{{{\rm{Var}}}}({\hat{N}}_{n})$ for a purely uncorrelated normalization. Here, *m*, *n* index over all pairs of frequency modes {*ω*_*m*_, *ω*_*n*_}. Observe that the gain-optimized NRF (${{\min }_{{g}_{mn}}}\,{{{{\rm{NRF}}}}}_{mn}$) is not symmetric.

We note here that sometimes squeezing emerges in the photon flux observable but not in the intensity observable—this is attributed to the significant difference in photon energy across the different combs (see SI for an investigation of this effect for a simple 3-comb system). The cascading nonlinear processes introduce strong quantum correlations in excess of 10 dB below the uncorrelated limit that span the entire multi-comb system, along with several low noise states exceeding 10 dB below the corresponding shot noise limit. Importantly, because the combs can lie at very different frequencies, our mechanism can introduce strong quantum correlations across very different spectral bands, including between combs that do not directly couple through the nearest-neighbor nonlinear interaction. This suggests that the idler comb mediates quantum correlations spanning the entire multi-comb system.

### Entanglement and cluster state generation

Multiple metrics have been put forth to assess CV entanglement in multimode systems^40–43^. Here, we use the positive partial transpose (PPT) criterion and the associated logarithmic negativity^40^, which assess the entanglement of an arbitrary bipartition of a system of *N*_tot_ modes through violation of the Robertson-Schrödinger (RS) uncertainty principle. This criterion is a sufficient condition for entanglement between the two partitions. One can look at the symplectic eigenvalues $\tilde{\nu }$ of the partially transposed covariance matrix and show violation of the RS principle through the condition ${\tilde{\nu }}_{\min } < 1$, where ${\tilde{\nu }}_{\min }$ is the minimum symplectic eigenvalue (see Methods for more details).

**Fig. 3:**
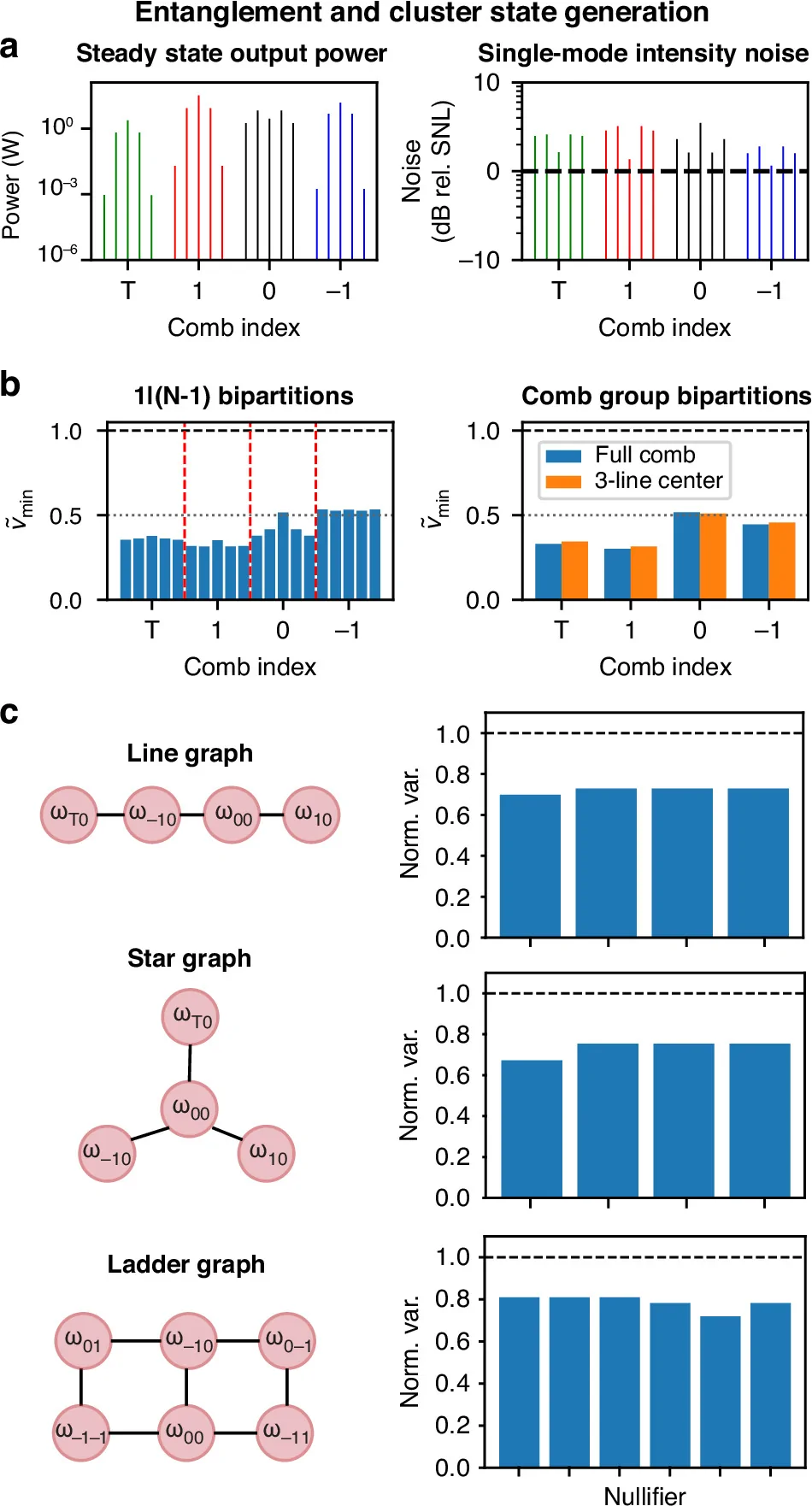
Entanglement and cluster state generation. **a** Steady state output power and intensity noise for all modes. **b** Minimum symplectic eigenvalue ${\tilde{\nu }}_{\min }$ for 1∣(*N*_tot_ − 1) (where *N*_tot_ denotes the total number of modes in the system) bipartitions and bipartitions between each comb (or its central subset of lines) and the rest of the system. **c** Cluster state generation with line, star, and ladder graphs. We assume 5% optical outcoupling loss, 10% idler outcoupling loss, and 1% intrinsic loss for all modes. Pumping strengths are 40 W for the pump comb and 7 W for the seed comb (same spectral profile as Fig. 1). For the ladder graph only, the pump and seed power are both 400 W, with 25% outcoupling loss. Stronger driving and smaller *μ*/*γ* allowed all six ladder nullifiers to drop below the vacuum level

In Fig. 3, we study the presence of spectrally addressable entanglement in the multi-comb system. Figure 3a presents a 4-comb system where all modes have intensity noise above the shot noise limit. In Fig. 3b, we show how two types of bipartitions, namely those of the form 1∣(*N*_tot_ − 1) (entanglement between one comb line and the rest of the system) and “comb group” bipartitions (entanglement between an entire comb or its central subset of lines and the rest of the system), show ${\tilde{\nu }}_{\min }$ well below the PPT threshold for entanglement ${\tilde{\nu }}_{\min }=1$.

We next consider entanglement in the form of cluster state generation. We search for 4-mode line- and star-cluster candidates by scanning all 4-mode subsets of the central-line modes, extracting each candidate covariance submatrix, and optimizing local phase rotations together with an overall edge-weight scale to minimize the normalized graph-nullifier variances. Candidates are ranked by a cost function combining the worst and mean normalized nullifier noise, and the best-ranked subset for each graph topology is identified. Gaussian PPT tests over all unique bipartitions are also used as supplementary entanglement diagnostics. More details about the cluster state identification are provided in the SI. Figure 3c demonstrates how both graphs are accompanied by nullifier variances below the unity-normalized vacuum reference, indicating that the selected modes simultaneously realize the graph correlations expected of two different types of finite-squeezing CV cluster states. Here, each nullifier is associated with a node in the target graph. For a target graph, the nullifiers are defined as ${\hat{\delta }}_{j}={\hat{p}}_{j}-g{\sum }_{k}{A}_{jk}{\hat{x}}_{k}$ where *A* denotes the target graph adjacency matrix, *g* is the fitted edge-weight scale, and ${\hat{p}}_{j},{\hat{x}}_{j}$ are the momentum and position quadratures for each mode in the graph.

To highlight the genuinely two-dimensional structure of the state space (the inter- and intracomb dimensions), a ladder-graph subset was identified on a 2 × 3 patch of modes indexed by subcomb number and intracomb line number (Fig. 3c). All optimized nullifier variances for this ladder candidate fall below the corresponding vacuum reference, indicating a finite-squeezing CV cluster-state structure on a graph that explicitly requires both synthetic frequency axes. The line and star graphs demonstrate graph reconfigurability, whereas the ladder graph demonstrates use of the full two-dimensional synthetic frequency lattice, highlighting the potential of using multiple synthetic dimensions for multi-dimensional cluster state generation.

We note that recent microcomb work has already demonstrated frequency-domain CV cluster states within a single Kerr resonator mode family^13^. Our contribution here is different: we target bright, multispectral cluster states formed by idler-mediated coupling between distinct subcombs, enabling entanglement resources whose nodes are separated not only in mode index but also in spectral function, with natural use cases in transduction and spectrally separated sensing. The entanglement resource we propose here is naturally framed around bright output carriers that act as physically different spectral channels.

Finally, we also note that sub-shot noise nullifier variances do not prove genuine multipartite entanglement. We show that genuine tripartite entanglement by the van Loock-Furusawa criterion^42^ is achievable in the SI.

### Covariance matrix engineering

We next explore the possibility of engineering the covariance matrix to optimize a target metric, such as squeezing between two selected modes. Three tools to do so are dissipation, pump, and dispersion engineering. Dissipation engineering enables control over the loss (*Q* factor) experienced by each frequency mode and the interplay between loss and nonlinear coupling enables one to shape nonlinear energy flow in the multi-comb system, affecting both mean field and quantum noise behavior. Through pump pulse engineering, one can control how strongly different modes in the system are excited. Finally, through dispersion engineering, one can tailor the nonlinear interaction strength for the three-wave mixing processes through tunable phase matching.

**Fig. 4:**
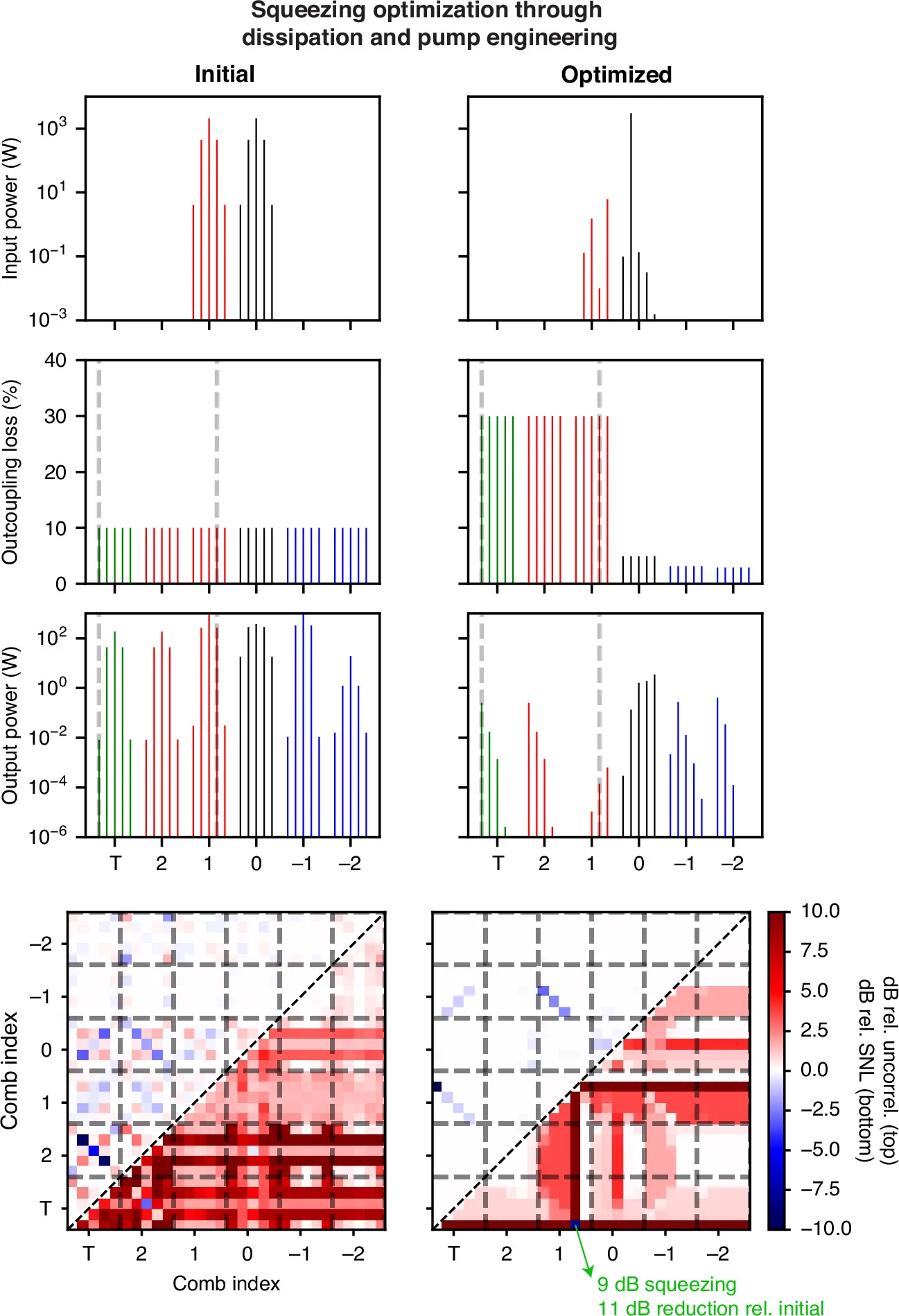
Squeezing optimization through dissipation and pump engineering. Optimization of “remote pair” two-mode squeezing between a mode in the mid-IR idler comb and a mode in one of the optical combs. Shown are the initial and optimized pump and seed profiles, loss profiles, mean field distributions, and gain-optimized pairwise photon flux noise heatmaps. Initial (baseline) parameters: 5% roundtrip intrinsic loss for all modes, 10% roundtrip outcoupling loss for all modes, maximum pump power 1 kW, and maximum seed power 1 kW. The optimization is over the outcoupling losses of the subcombs and the pump/seed profile, subject to the constraint that the total pump and seed power should not exceed the initial values. Note that the optimizer raises outcoupling loss, which is consistent with the escape-efficiency argument for generating squeezing described in the caption of Fig. 2

In Fig. 4, we explore a simple example of covariance matrix optimization using the first two of the aforementioned tools (we explore the role of modified phase matching by changing *ω*_*m*_ in the SI). The objective in this optimization is to maximize “remote pair” squeezing between a mode in the mid-IR idler comb and a mode in an optical comb. The optimizable parameters are the losses for each comb (assumed constant across the comb given the small frequency span relative to the intercomb spacing) and the pump/seed comb power distribution across their comb lines (achievable with spectral pulse shaping). The optimization is constrained to keep the target modes “bright” (i.e. above a certain threshold power) and to not exceed the initial total pump and seed power. Further details about the optimization and examples of other optimizations with different objectives are presented in the SI. Here, optimization of the remote pair objective achieves 9 dB squeezing below the shot noise limit (and 11 dB below the initial baseline).

These results, while simple, showcase the promise of using dissipation and pump engineering for shaping the quantum noise and covariance matrix of multimode nonlinear systems. Although considerable work has explored the application of these and similar tools to the mean field distributions and classical dynamics of multimodal systems^44–48^, their application to generating tailored multimode quantum states has remained until now unexplored.

The forms of pump and dissipation engineering we consider here are readily achievable experimentally. The former is most directly controlled by changing the modulation waveform applied to the quasi-CW pump/seed, or by performing pulse shaping if broadband pulses are utilized, as considered in the next section^49^. The latter can be obtained from judicious choice of optical elements with frequency-dependent loss, such as dichroic mirrors, spectral-to-spatial intracavity modulation, or etalons/Bragg gratings/high-finesse Fabry-Perot resonators (for individual frequency mode control).

### Broadband quantum correlations through cascaded ultrafast pulse generation

Frequency combs are also commonly generated through mode-locked lasers^50–52^, where the Fourier transform of a pulse train with repetition rate *f*_*m*_ = *ω*_*m*_/2*π* corresponds to a frequency comb with spacing *f*_*m*_. To complement our discrete mode analysis above, in this section, we calculate the quantum correlations in a multimode nonlinear system that supports cascaded SFG and DFG processes and is pumped by ultrafast pulses. Here, we simulate ultrafast pulses propagating through a length *L*_cry_ of a nonlinear crystal supporting the cascaded second-order nonlinear processes that form the basis of our work. Simulating a (phase-locked) pulse train would discretize the broadband pulse spectra into comb lines to realize a true frequency comb. In addition to second-order nonlinearity, we include the effects of Kerr nonlinearity, self-steepening, and dispersion (using the frequency-dependent refractive index of lithium niobate^53^), as detailed in the SI. The basis for our classical simulations is a system of coupled generalized nonlinear Schrödinger equations (GNLSEs), each equation describing the propagation of an individual pulse through the nonlinear medium. The GNLSE is a standard model for simulating ultrafast pulse propagation in systems with dispersion and nonlinearity, such as optical fibers^54–56^.

Our simulated system can represent several realistic experimental platforms, including both single-pass (where each pump pulse interacts with the nonlinear crystal only once) and multi-pass configurations. The latter allows smaller nonlinear crystal lengths to be used while still retaining strong nonlinearity, and can take the form of a synchronously pumped optical parametric oscillator^57^ or a configuration similar to a regenerative parametric amplifier^58^, where the pulses circulate in a cavity multiple times before being outcoupled. The regenerative-amplifier-like configuration forms the basis of our simulations here.

**Fig. 5:**
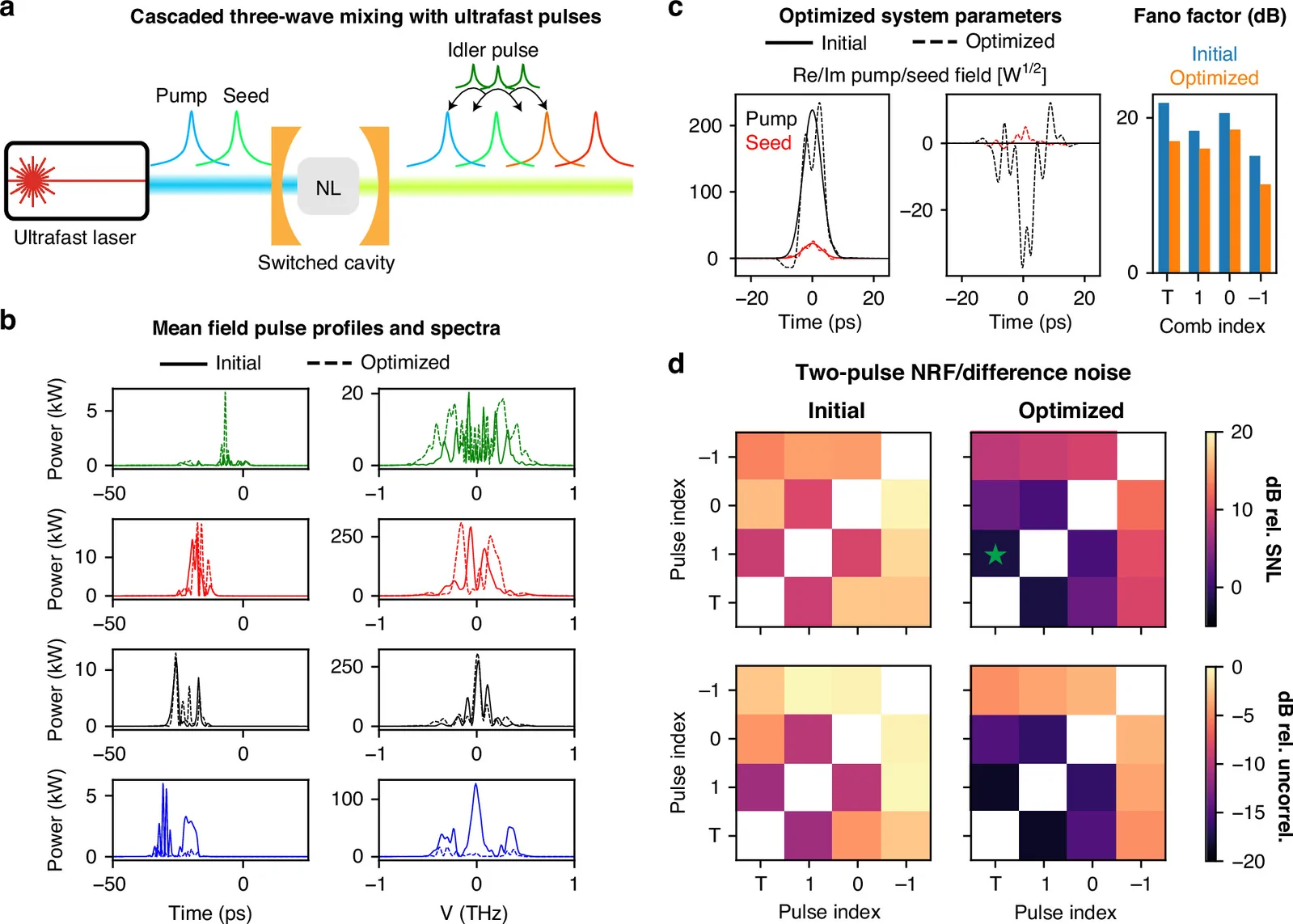
Quantum correlations in an ultrafast system with cascaded three-wave mixing processes. **a** Ultrafast laser pumping a *χ*^(2)^ nonlinear cavity phase matched for cascaded DFG and SFG. **b** Mean field spectra and temporal pulse profiles for each pulse, where green denotes the idler pulse and red, black, and blue the optical pulses in order of increasing frequency. **c** Pump/seed pulse shaping to optimize the noise reduction factor (NRF) between two selected pulses. Also shown is the high intensity noise of the individual pulses, typical of high-power systems. **d** Gain-optimized NRF heatmaps normalized to uncorrelated sum noise and shot noise. The green star indicates the target pair for squeezing. This optimization achieves 2 dB squeezing below the SNL and 11 dB reduction relative to the initial NRF. Pump wavelength: *λ*_0_ = 465 nm, idler wavelength: *λ*_*T*_ = 4.07 μm. Simulated nonlinear strengths are second-order nonlinearity *d*_eff_ = 16 pm/V and third-order (Kerr) nonlinearity *n*_2_ = 3 × 10^−19^ m^2^/W. Dispersion was modeled through the refractive index profile of lithium niobate^53^. Pump pulse parameters: peak power 50 kW, pulse duration 5.0 ps. Seed pulse parameters are identical, except with peak power 500 W. The nonlinear crystal length is 2 mm, and 10 roundtrips in a 1 m long cavity are simulated. 2% outcoupling loss and 5% intrinsic loss for all modes are assumed

In Fig. 5b, we calculate the mean-field spectra and pulse profiles for the system schematically shown in Fig. 5a by propagating the coupled nonlinear system of *I* + 1 GNLSEs (*I* subcombs and the idler comb) forward for a length *L*_cry_ = 2 mm in the nonlinear crystal, applying a fixed attenuation (coming from mirror loss), and repeating for a fixed number of roundtrips.

To calculate quantum statistics in the ultrafast system we consider here, we use quantum sensitivity analysis, a recently developed method for efficiently calculating fluctuations in observables of a multimode nonlinear system from the Jacobian (“sensitivity”) of a single classical simulation of the system’s forward dynamics and any noise channels entering the system^59,60^. As described in the SI, we calculate the covariance matrix of the system from the Jacobian of the nonlinear interaction as well as from the loss channels in the cavity. From the covariance matrix, we can calculate the technologically relevant noise reduction factor (NRF) given by Eq. (2). In this system, *N*_*m*_ denotes the grouped time-bin or frequency-bin integrated photon number in window *m* (which sweeps over all of the pulses in the system). Because the sensitivity analysis linearizes about the actual classical trajectory rather than a fixed point, steady state is not required; reported NRFs correspond to the state after the simulated round trips.

In Fig. 5b–d, we perform an optimization to concentrate squeezing between the mid-IR pulse and an optical pulse. The optimizable parameters include the pump and seed pulse profiles, the pump-seed delay, and the seed pulse energy (constrained to not exceed the initial baseline value). Details about the optimization protocol are provided in the SI. Figure 5b plots the initial and optimized temporal and spectral profiles for each pulse, while Fig. 5c shows the optimized pump and seed fields from the pulse shaping optimization. The same panel also shows the Fano factor, defined as ${F}_{i}={{{\rm{Var}}}}({\hat{N}}_{i})/\langle {\hat{N}}_{i}\rangle$, where ${{{\rm{Var}}}}({\hat{N}}_{i}),\langle {\hat{N}}_{i}\rangle$ denote the variance and mean photon number for the *i*’th pulse, respectively. A shot noise limited pulse corresponds to *F*_*i*_ = 1. Finally, Fig. 5d plots the initial and optimized NRF matrices. The NRF maps are constructed with a single bin encompassing each pulse in the system, thereby probing whole-pulse interpulse correlations. The pulse shaping serves to selectively enhance nonlinear channels that maximize correlations between the two selected pulses for the optimization, namely the mid-IR idler pulse (wavelength 4.07 μm) and the optical pulse at 525 nm. The result is squeezing of 2 dB below the SNL and 11 dB below the initial NRF, despite Fano factors of the pulses over 10 dB above the SNL.

## Discussion

In this work, we showed how cascaded three-wave mixing mediated by a common idler comb can generate strong inter- and intracomb quantum correlations across multiple frequency combs. We further showed how covariance-matrix engineering can concentrate squeezing into selected mode pairs or shape the correlations toward target multipartite structures.

The key distinction of this work is not simply broader spectral coverage, but a source architecture in which a common idler comb coherently couples several distinct nondegenerate combs. The resulting resource contains both inter- and intracomb correlations among bright, spectrally resolved output modes. In contrast to much of the quantum comb literature, which has emphasized below-threshold squeezed-supermode physics, the present setting is naturally expressed in the emitted mode basis itself and is therefore well matched to applications requiring brightness and direct spectral addressability.

Classical frequency comb platforms have primarily been developed as coherent spectral grids, with emphasis on bandwidth, conversion efficiency, repetition rate, linewidth, stability, tunability, and integration^61–65^. Classical dual comb, interleaved comb, and coupled resonator systems already exploit multiple combs for heterodyne spectroscopy, ranging, communications, and efficient signal generation^66^. Our distinction is therefore not simply the use of multiple combs or a larger total bandwidth. Rather, distinct nondegenerate combs are coherently linked by a common idler comb, so that the output forms a single hierarchical quantum resource with both intracomb and intercomb correlations. Recent quantum microcomb work has emphasized multi-wavelength entanglement and scaling to many quantum channels from a resonator^66^; our architecture complements this direction by emphasizing controllable correlations across separate comb families while retaining direct frequency-mode addressability. The SI provides a more detailed comparison with related classical and quantum comb architectures.

Access to bright quantum light directly in the frequency-mode basis makes the platform attractive for multispectral sensing. In particular, one can probe a sample in a wavelength range suited to the material response while performing readout in a spectral channel with more favorable detection technology. In the SI, we illustrate this idea for outside-cavity remote loss sensing and intracavity transduction sensing, where the predicted sensitivities surpass direct-detection shot-noise limits.

We also outlined experimental routes in both quasi-CW and ultrafast settings. In the ultrafast case, true steady-state multi-comb operation would require a synchronously pumped multimode cavity. Our simulations are primarily intended to describe a free-space cavity with periodically poled lithium niobate, and the SI gives realistic parameter estimates for that platform. Longer term, an important direction is the translation of these ideas to monolithic and integrated comb and optical parametric oscillator (OPO) architectures. Exciting progress toward integrated and monolithic frequency combs and ultrafast OPOs has been made^33,47,63,67,68^ and we envision that these platforms can already realize some of the physics described here.

Our mechanism realizes coupled frequency combs whose intercomb spacing *ω*_*T*_ far exceeds the intracomb spacing *ω*_*m*_. In practice, *ω*_*m*_ may lie at MHz or GHz frequencies, while *ω*_*T*_ can lie at microwave, terahertz, or infrared frequencies. Here, we have focused on the last regime, as it enables us to generate combs in highly disparate spectral ranges, but we emphasize that the physical mechanism holds irrespective of *ω*_*m*_ and *ω*_*T*_. This therefore admits a wide range of physical platforms (from optomechanical and microwave resonators to optical comb systems) to realize the hierarchical frequency comb architecture.

Although our emphasis here is on directly usable correlations in the emitted frequency basis, the same states could also be interfaced with programmable linear optical processors that identify or synthesize useful modal bases, including ones that concentrate squeezing into selected collective modes^69–71^. This suggests a natural intersection between hierarchical multi-comb sources and reconfigurable photonic processors.

Beyond the quantum-optical regime studied here, this platform may also offer a route to synthetic-frequency physics in two coupled frequency dimensions^72–79^. In our system, the intracomb and intercomb frequency axes provide a natural hierarchical two-dimensional synthetic space. This raises the possibility that suitably engineered modulation, dissipation, or coupling could be used to realize topological and non-Hermitian band structures in multimode quantum comb settings, including states distributed across distinct but coupled combs.

Overall, our results establish a framework for quantum correlations in hierarchical multi-comb systems generated by cascaded three-wave mixing. Beyond extending comb bandwidth, this architecture creates a bright multipartite resource distributed across distinct but coupled spectral structures, with clear relevance to multispectral sensing and spectrally multiplexed continuous-variable quantum photonics.

## Materials and methods

### Heisenberg equations of motion

For the discrete frequency comb systems considered in much of the main text, we use the Heisenberg-Langevin formalism to analyze mean field and noise properties. The mean field equations are given by Eq. 1 in the main text, which are coupled mode equations that include driving, dissipation, and the allowable three-wave mixing processes. These equations can easily be derived from the nonlinear Hamiltonian (*ℏ* = 1)

3 $${{{\mathcal{H}}}} =\sum\limits_{j}{\omega }_{j}^{T}{\hat{a}}_{j}^{T{{\dagger}} }{\hat{a}}_{j}^{T}+\sum\limits_{ij}{\omega }_{ij}{\hat{a}}_{ij}^{{{\dagger}} }{\hat{a}}_{ij}\\ +\sum\limits_{ijk}{{{\rm{i}}}}{\beta }_{ijk}{\hat{a}}_{-k}^{T{{\dagger}} }{\hat{a}}_{ij}^{{{\dagger}} }{\hat{a}}_{i-1,j-k}+{{{\rm{h.c.}}}}$$

where the first two terms represent the bare Hamiltonians for all of the frequency modes in the system and the third term encompasses all of the cascaded three-wave mixing processes. For brevity, we have omitted driving and dissipation terms from the Hamiltonian (dissipation makes ${{{\mathcal{H}}}}$ non-Hermitian) and add them directly to the equations of motion.

Moving into the interaction picture and linearizing these equations about the mean field steady state and adding appropriate Langevin force noise terms, we have

4 $$\delta {\dot{\hat{a}}}_{-k}^{T} =	\sum\limits_{ij}[{\beta }_{ijk}({a}_{ij}^{* }\delta {\hat{a}}_{i-1,j-k}+\delta {\hat{a}}_{ij}^{{{\dagger}} }{a}_{i-1,j-k})]\\ 	-({\gamma }_{T}+{\mu }_{T})\delta {\hat{a}}_{-k}^{T}+{\hat{F}}_{-k}^{T} \\ \delta\dot {\hat{a}}_{ij} =	\sum\limits_{k}-{\beta }_{i+1,j+k,k}^{* }(\delta {\hat{a}}_{-k}^{T}{a}_{i+1,j+k}+{a}_{-k}^{T}\delta {\hat{a}}_{i+1,j+k})\\ 	+\sum\limits_{k}{\beta }_{ijk}(\delta {\hat{a}}_{-k}^{T{{\dagger}} }{a}_{i-1,j-k}+{a}_{-k}^{T* }\delta {\hat{a}}_{i-1,j-k})\\ 	-({\gamma }_{ij}+{\mu }_{ij})\delta {\hat{a}}_{ij}+{\hat{F}}_{ij}$$

where the Langevin forces are zero mean and satisfy the correlators^80,81^

5 $$\langle {\hat{F}}_{-k}^{T}(t){\hat{F}}_{-{k}^{{\prime} }}^{T{{\dagger}} }({t}^{{\prime} })\rangle =	2({\gamma }_{T}+{\mu }_{T})(1+{n}_{-k}^{T,{{{\rm{th}}}}}){\delta }_{k{k}^{{\prime} }}\delta (t-{t}^{{\prime} })\\ \langle {\hat{F}}_{-k}^{T{{\dagger}} }(t){\hat{F}}_{-k^{\prime} }^{T}({t}^{{\prime} })\rangle =	2({\gamma }_{T}+{\mu }_{T}){n}_{-k}^{T,{{{\rm{th}}}}}{\delta }_{kk^{\prime} }\delta (t-t^{\prime} )\\ \langle {\hat{F}}_{ij}(t){\hat{F}}_{i^{\prime} j^{\prime} }^{{{\dagger}} }({t}^{{\prime} })\rangle =	2({\gamma }_{ij}+{\mu }_{ij})(1+{n}_{ij}^{{{{\rm{th}}}}}){\delta }_{i{i}^{{\prime} }}{\delta }_{jj^{\prime} }\delta (t-t^{\prime} )\\ \langle {\hat{F}}_{ij}^{{{\dagger}} }(t){\hat{F}}_{{i}^{{\prime} }{j}^{{\prime} }}({t}^{{\prime} })\rangle =	2({\gamma }_{ij}+{\mu }_{ij}){n}_{ij}^{{{{\rm{th}}}}}{\delta }_{i{i}^{{\prime} }}{\delta }_{jj^{\prime} }\delta (t-t^{\prime} )$$

which relates the Langevin noise correlators to the dissipation rates in the system. Here, ${n}_{-k}^{T,{{{\rm{th}}}}}={\left(\exp (\hslash {\omega }_{-k}^{T}/{k}_{B}T)-1\right)}^{-1},{n}_{ij}^{{{{\rm{th}}}}}={\left(\exp (\hslash {\omega }_{ij}/{k}_{B}T)-1\right)}^{-1}$ are the Bose thermal occupation numbers. For the examples we consider, $\hslash {\omega }_{-k}^{T},\hslash {\omega }_{ij}\gg {k}_{B}T$ at room temperature, so we will take ${n}_{-k}^{T,{{{\rm{th}}}}},{n}_{ij}^{{{{\rm{th}}}}}\to 0$.

To calculate output noise, we use temporal coupled mode theory to write the input-output relations

6 $$\delta {\hat{s}}_{k,{{{\rm{out}}}}}=\sqrt{2{\gamma }_{k}}\delta {\hat{a}}_{k}-\delta {\hat{s}}_{k}$$

where for a coherent drive $\langle \delta {\hat{s}}_{k}(t)\delta {\hat{s}}_{k}^{{{\dagger}} }(t^{\prime} )\rangle =\delta (t-t^{\prime} )$ and $\langle \delta {\hat{s}}_{k}(t){\hat{F}}_{k}^{{{\dagger}} }(t^{\prime} )\rangle =\langle {\hat{F}}_{k}(t)\delta {\hat{s}}_{k}^{{{\dagger}} }(t^{\prime} )\rangle =\sqrt{2{\gamma }_{k}}\delta (t-t^{\prime} )$.

From the Fourier transform of Eq. (4), the intracavity noise spectra can be calculated from

7 $$\left(\begin{array}{c}\delta {\hat{{{{\bf{a}}}}}}_{k}(\Omega )\\ \delta {\hat{{{{\bf{a}}}}}}_{k}^{{{\dagger}} }(\Omega )\end{array}\right)={{{{\bf{M}}}}}^{-1}(\Omega )\left(\begin{array}{c}{{{\bf{F}}}}(\Omega )\\ {{{{\bf{F}}}}}^{{{\dagger}} }(\Omega )\end{array}\right)$$

where **M**^−1^(*Ω*) denotes the inverse of the fluctuation matrix at noise frequency *Ω* and can be calculated from the steady state field values. In combination with Eq. (6), we can then immediately calculate the output noise spectra $\langle \delta {\hat{s}}_{k,{{{\rm{out}}}}}(\Omega )\delta {\hat{s}}_{k,{{{\rm{out}}}}}^{{{\dagger}} }(\Omega )\rangle$. All noise statistics in the main text are calculated at 1 MHz noise frequency.

To validate the stability of the steady state solution (and therefore our ability to calculate noise properties by linearization), one can compute the eigenvalues of  − **M**(*Ω* = 0), which represents the Jacobian of the system. If all eigenvalues have negative real part (as they are for examples in the main text), the steady state is stable. Equivalently, one can compute the Bogoliubov linear stability matrix **G** of the system, defined as

8 $$\frac{d}{dt}\left(\begin{array}{c}\delta {{{\bf{a}}}}\\ \delta {{{{\bf{a}}}}}^{* }\end{array}\right)={{{\bf{G}}}}\left(\begin{array}{c}\delta {{{\bf{a}}}}\\ \delta {{{{\bf{a}}}}}^{* }\end{array}\right)+{{{\bf{F}}}}(t)$$

and ensure all eigenvalues have negative real part.

### Peres-Horodecki/PPT criterion

We describe the output fluctuations using dimensionless quadratures

$${\hat{x}}_{j}={\hat{A}}_{j,{{{\rm{out}}}}}+{\hat{A}}_{j,{{{\rm{out}}}}}^{{{\dagger}} },\qquad {\hat{p}}_{j}=-i({\hat{A}}_{j,{{{\rm{out}}}}}-{\hat{A}}_{j,{{{\rm{out}}}}}^{{{\dagger}} })$$

where ${\hat{A}}_{j,{{{\rm{out}}}}}$ is a normalized output-mode operator corresponding to ${\hat{s}}_{j,{{{\rm{out}}}}}$. We collect the quadratures into the vector

$$\hat{{{{\bf{X}}}}}={({\hat{x}}_{1},{\hat{p}}_{1},{\hat{x}}_{2},{\hat{p}}_{2},\ldots ,{\hat{x}}_{{N}_{{{{\rm{tot}}}}}},{\hat{p}}_{{N}_{{{{\rm{tot}}}}}})}^{T}$$

The output-field fluctuation covariance matrix at the selected analysis frequency is defined by

$${V}_{mn}=\frac{1}{2}\left\langle \Delta {\hat{X}}_{m}\Delta {\hat{X}}_{n}+\Delta {\hat{X}}_{n}\Delta {\hat{X}}_{m}\right\rangle$$

where $\Delta \hat{{{{\bf{X}}}}}=\hat{{{{\bf{X}}}}}-\langle \hat{{{{\bf{X}}}}}\rangle$. Because the quadratures are defined without a factor of $1/\sqrt{2},[{\hat{x}}_{j},{\hat{p}}_{k}]=2i{\delta }_{jk}$ and therefore

$$[\hat{{{{\bf{X}}}}},{\hat{{{{\bf{X}}}}}}^{T}]=2i{{{\bf{W}}}},\qquad {{{\bf{W}}}}= {\bigoplus}_{j = 1}^{{N}_{{{{\rm{tot}}}}}}\left(\begin{array}{rc}0&1\\ -1&0\end{array}\right)$$

Physical Gaussian states obey the Robertson-Schrödinger uncertainty relation

9 $${{{\bf{V}}}}+i{{{\bf{W}}}} \succeq 0$$

Equivalently, all symplectic eigenvalues satisfy *ν*_*k*_≥1. The symplectic eigenvalues are obtained from the positive moduli of the eigenvalues of *i***WV**.

For a specified bipartition, partial transposition is implemented by reversing one quadrature of every mode in one partition^82^. In the present convention this corresponds to ${\hat{p}}_{n}\to -{\hat{p}}_{n}$ If $\widetilde{{{{\bf{V}}}}}$ denotes the resulting partially transposed covariance matrix and

10 $${\widetilde{\nu }}_{\min } < 1$$

then the state has a negative partial transpose and is therefore entangled across that bipartition.

For Gaussian states, PPT violation is necessary and sufficient for entanglement across 1 × (*N*_tot_ − 1) bipartitions^83^. For more general *M* × (*N*_tot_ − *M*) bipartitions, PPT violation remains sufficient to establish entanglement, but the absence of PPT violation does not generally prove separability. We also calculate the logarithmic negativity^84^,

11 $${E}_{{{{\mathcal{N}}}}}={\sum}_{k}\max \left(0,-{\log }_{2}{\widetilde{\nu }}_{k}\right)$$

The minimum partially transposed symplectic eigenvalue is therefore an entanglement diagnostic, while ${E}_{{{{\mathcal{N}}}}}$ incorporates all partially transposed symplectic eigenvalues below the separability threshold.

## Supplementary information

Supplementary Information for: Long-range spectral entanglement and quantum correlations in highly nondegenerate multi-comb and ultrafast systems

## Data Availability

All data presented in this work is reproducible from code available from the authors on reasonable request.
