# Supplementary material for: Long-range spectral entanglement and quantum correlations in highly nondegenerate multi-comb and ultrafast systems
Source: Light Sci Appl. 2026 Nov 5;15:394. doi: 10.1038/s41377-026-02491-3 (PMC13736843; doi:10.1038/s41377-026-02491-3)
Supplement: Supplementary file 1 [file 41377_2026_2491_MOESM1_ESM.pdf]

Supplementary Information for: Long-range spectral entanglement and quantum correlations in highly nondegenerate multi-comb and ultrafast systems

Sahil Pontula[1,2,3,*], Debasmita Banerjee[4], Marin Soljačić[1,3], and Yannick Salamin[4,*]

[1]*Department of Physics, MIT, Cambridge, MA 02139, USA.*

[2]*Department of Electrical Engineering and Computer Science, MIT, Cambridge, MA 02139, USA.*

[3]*Research Laboratory of Electronics, MIT, Cambridge, MA 02139, USA.*

[4]*CREOL, The College of Optics and Photonics,*

University of Central Florida, Orlando, Florida 32816, USA.

* *Contact: spontula@mit.edu, yannick.salamin@ucf.edu.*

Abstract

In this Supplementary Information (S.I.), we present derivations of results reported in the main text, further details about the systems considered, and supplemental figures.

Contents

A. Cluster states and multipartite entanglement 4

A.1. Genuine multipartite entanglement 5

A.2. Detection 6

B. Three comb system 7

C. Spectroscopy/sensing applications 7

C.1. Outside-cavity remote loss sensing 7

C.2. Intracavity transduction sensing 10

D. Coupled generalized nonlinear Schrödinger equations 11

E. Ultrafast mean-field and noise propagation model 12

F. Covariance matrix optimization procedure 14

F.1. Discrete entanglement model 14

F.2. Ultrafast pulse model 16

G. Other optimization examples 18

G.1. Remote loss sensing optimization 18

G.2. Cluster state optimization 18

H. Design considerations and estimation of experimental parameters 18

H.1. Second order nonlinearity and phase matching 20

H.2. Intracomb spacing 22

H.3. Pump strengths and noise 23

H.4. Losses 25

H.5. Pump and dissipation engineering 28

H.6. Comb stability 29

H.7. Other materials 29

I. Comparison to prior works 30

References 32

A. CLUSTER STATES AND MULTIPARTITE ENTANGLEMENT

We begin with a description of the procedure for identifying 4-node graphs. Starting from the output Gaussian covariance matrix of the full multimode system, we restricted the cluster state search to the central spectral line of each comb, i.e. one mode from each of the $I + 1$ comb families. For each target graph topology, e.g., a 4-node line graph and a 4-node star graph, we enumerated all 4-mode subsets drawn from this candidate set and all inequivalent assignments of the selected physical modes to the graph vertices. For the line graph, all mode orderings were tested up to reversal symmetry; for the star graph, only inequivalent choices of the hub mode were distinguished, since permutations of the leaves are redundant.

For each ordered 4-mode candidate, we extracted the corresponding 8×8 covariance submatrix in $(\hat{x}_1, \hat{p}_1, ..., \hat{x}_4, \hat{p}_4)$ ordering and optimized over independent local phase rotations $\{\theta_j\}$ applied to each mode. After these rotations, we defined the graph nullifiers as

$$\hat{\delta}_j = \hat{p}_j - g \sum_k A_{jk} \hat{x}_k \tag{S1}$$

where A is the adjacency matrix of the target graph and g is a fitted edge-weight scale. The phase angles and g were jointly optimized to minimize a cost function favoring uniformly small nullifier noise,

$$\mathcal{L} = \max_j \left(\frac{\mathrm{Var}(\hat{\delta}_j)}{\mathrm{Var}_{\mathrm{vac},j}} \right) + 0.15 \mathrm{mean}_j \left(\frac{\mathrm{Var}(\hat{\delta}_j)}{\mathrm{Var}_{\mathrm{vac},j}} \right) \tag{S2}$$

where the prefactor 0.15 is an empirically chosen small weight to break ties toward uniformly small nullifiers. The vacuum baselines are

$$\mathrm{Var}_{\mathrm{vac},j} = 1 + g^2 \sum_k A_{jk}^2 \tag{S3}$$

The optimization was carried out with a bounded Powell search using several deterministic and random initializations. All candidate subsets were then ranked by the optimized objective value, and the best-ranked subset was reported as the strongest 4-mode line-cluster or star-cluster candidate. As an additional diagnostic, each optimized 4-mode candidate was also tested for bipartite inseparability using the Gaussian PPT criterion across all unique $1|3$ and $2|2$ bipartitions. These

PPT values were stored as supporting entanglement diagnostics, but the subset ranking itself was determined by the optimized nullifier objective.

For the ladder states, the search is not over arbitrary modes from the full device. Instead, we first define a centered 2×3 rectangular optical patch spanning the comb modes, which picks two neighboring optical subcombs and the three central spectral lines within them. This gives a fixed six-mode candidate set arranged as a two-row, three-column lattice. We then target the adjacency matrix of a 2×3, i.e. a ladder graph with horizontal rails and vertical rungs. Because the candidate patch already contains exactly six modes, the ladder search evaluates that one physical six-mode subset under all assignments of the six modes to the six graph vertices. For each assignment, it extracts the 12×12 covariance submatrix, applies the same local phase optimization and fitted global edge-weight scale g, and minimizes the same normalized nullifier objective as above. The best assignment is reported as the ladder-state candidate, and a full Gaussian PPT scan over the nonredundant bipartitions of that six-mode subset is stored as a secondary entanglement diagnostic.

A.1. Genuine multipartite entanglement

Sub-shot noise nullifier variances do not by themselves indicate genuine multipartite entanglement. We show in this section tripartite entanglement certification for a 3-node line.

To complement the graph-conditioned nullifier analysis, we also evaluated a tripartite van Loock-Furusawa (vLF) witness [S1] on a three-mode subsystem shown. For a triplet of modes $(1, 2, 3)$, we define the three witness terms

$$\begin{aligned} B_{12} &= \mathrm{Var}(\hat{x}_1 - \hat{x}_2) + \mathrm{Var}(\hat{p}_1 + \hat{p}_2 + g_3\hat{p}_3) \\ B_{23} &= \mathrm{Var}(\hat{x}_2 - \hat{x}_3) + \mathrm{Var}(\hat{p}_2 + \hat{p}_3 + g_1\hat{p}_1) \\ B_{13} &= \mathrm{Var}(\hat{x}_1 - \hat{x}_3) + \mathrm{Var}(\hat{p}_1 + \hat{p}_3 + g_2\hat{p}_2), \end{aligned} \tag{S4}$$

with the real gains g_k chosen to minimize the corresponding momentum-sum variance. In this form, the criterion

$$B_{12} + B_{23} + B_{13} < 8 \tag{S5}$$

Quantity	Value
Nullifier 1	0.476
Nullifier 2	0.214
Nullifier 3	0.476
Max nullifier	0.476
B_{12}	1.72
B_{23}	2.96
B_{13}	1.66
$\sum B$	6.34
GME threshold	8.0
Witness margin	1.66

TABLE S1: **Genuine tripartite entanglement in a bright 3-node line graph.** The nodes, in order, are the center frequency modes of the idler comb, the first optical comb, and the second optical comb (in order of increasing frequency). Roundtrip outcoupling losses for the optical combs in order of increasing frequency: 0.84, 0.92, 0.94, 0.99, 0.15. Idler roundtrip outcoupling loss: 0.90. Intrinsic optical roundtrip loss: 0.051, 0.051, 0.28, 0.99, 0.05. Intrinsic idler roundtrip loss: 0.05. Pump power: 300 kW, seed power: 78 kW.

is sufficient for genuine tripartite entanglement. Table S1 demonstrates that the subset of three center modes from the idler and first two optical combs (from a 5-comb system) forms a line graph with genuine multipartite entanglement by the vLF criterion.

A.2. Detection

Although the results in Fig. 3 of the main text suggest that entanglement permeates the multi-comb system, detection of the full set of $(I+1)\times J$ modes may be impractical. The more experimentally relevant observables are the lower-dimensional structures highlighted by the added cluster state analysis (Fig. 3), such as the central-line triplets and the 4- and 6-mode cluster candidates. These can be probed with substantially reduced overhead by targeting only the subset of modes entering the corresponding nullifier or PPT witness, rather than the entire mode space.

For the quasi-CW EO-modulated cavity architecture considered in the main text, a practical route is to derive the local-oscillator (LO) references from the same narrowband source used to generate the pump sidebands, so that the LO remains phase coherent with the cavity drive. After spectral demultiplexing of the output into the different comb bands, one can interfere the selected bands with matching LO tones. In the simplest implementation, this naturally supports the central-line measurements most relevant to the cluster state results.

The center-line LO in each band can serve as a reference not only for direct homodyne of the

central modes, but also for RF-resolved detection of nearby off-center teeth through their beat notes with the center line. In this way, multiple modes within a given comb can be accessed using a single optical LO per comb together with phase-coherent electronic demodulation, substantially reducing the experimental overhead relative to one-LO-per-mode tomography.

B. THREE COMB SYSTEM

In Fig. S1, we study the quantum optics of the simplest possible multi-comb system consisting of a single cascading step, where three-wave mixing occurs between a single pump, signal, and idler comb. For completeness, we plot both a quasi-degenerate case where $\omega_1 \approx \omega_T$ as well as a nondegenerate case where $\omega_T \ll \omega_1$. The difference in these two cases emerges in photon energy-sensitive measurements, such as measurements of power or intensity noise. For example, note the absence of two-mode squeezing (relative to shot noise) in the nondegenerate case in Fig. S1b. This is primarily due to the vast difference in signal and idler photon energy. A plot of the photon flux difference noise reveals equally strong squeezing compared to the quasi-degenerate case. Bipartite entanglement is quadrature sensitive and does not depend on photon energy, giving rise to nearly identical minimum symplectic eigenvalues for the quasi-degenerate and nondegenerate cases.

Even in the three comb case, one can see the effects of the idler-mediated three-wave mixing as comb lines further from the center get populated. This manifests in additional correlation pathways besides direct DFG (see for example the strong intracomb correlations).

C. SPECTROSCOPY/SENSING APPLICATIONS

We consider in this section two types of sensing applications – one based on intracavity sensing and the other based on outside-cavity remote loss sensing.

C.1. Outside-cavity remote loss sensing

To showcase a concrete application enabled by the multi-comb system, we consider using the system as a source and putting a sample in the probe arm after the comb generation, as shown

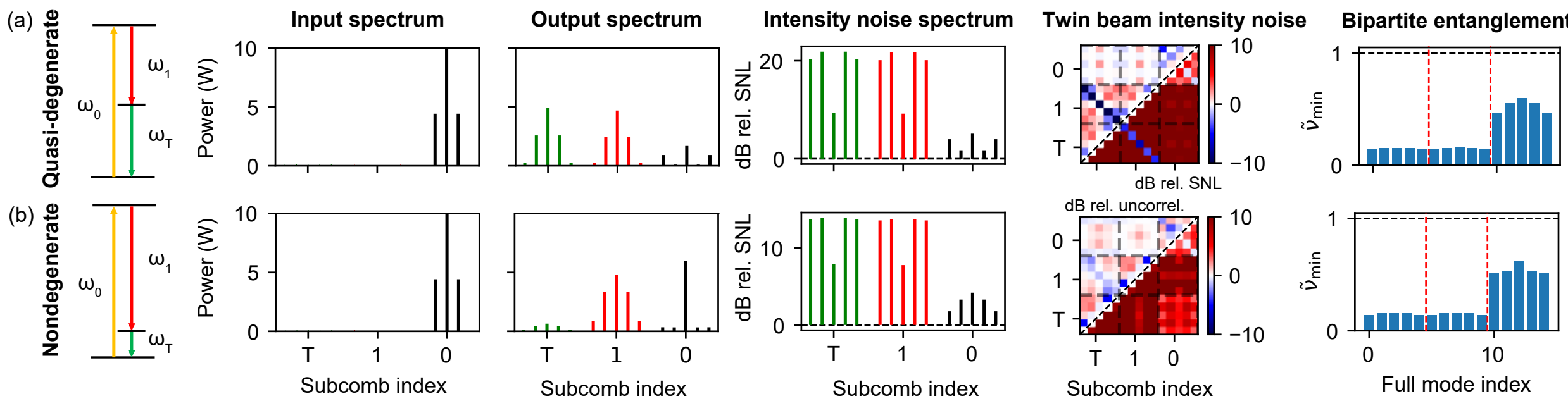

FIG. S1: **Quasi-degenerate and nondegenerate three comb systems.** (a) Quasi-degenerate three comb system with $\lambda_0 = 532$ nm, $\lambda_1 = 1063$ nm. (b) Nondegenerate three comb system with $\lambda_0 = 465$ nm, $\lambda_1 = 525$ nm. In both systems, $I = 2, J = 5$, crystal length $L_{\text{cry}} = 10$ mm, cavity length $L_{\text{cav}} = 10$ cm, outcoupling loss 5% for the optical modes and 10% for the idler modes, intrinsic cavity loss 0.5% for all modes, and $\beta_0 = 10^{-4}$ J$^{-1/2}$. The peak seed power is 1 mW and PPLN phase matched for DFG between the center comb lines is assumed. In the twin beam intensity (difference) noise heatmaps, the upper diagonal normalizes the noise to the uncorrelated sum noise, while the lower diagonal normalizes the noise to the two-mode shot noise limit. In the bipartite entanglement plots, we plot all "one versus rest" bipartitions $1|(N-1)$ where $N = (I+1) \times J$ is the total number of modes. The x axis denotes the mode in the single mode bipartition, with the red dashed lines demarcating the combs.

in Fig. S2a. To model ghost-spectroscopic readout, we add a weak frequency-dependent loss perturbation to one sample-coupled optical comb line and compute the response of a remote mid-IR idler line. We then compare the perturbation sensitivity of an optimized correlated two-line estimator against that of an uncorrelated spectral pair. This isolates the sensing benefit of the inter-comb covariance itself, rather than relying only on raw intensity-difference squeezing. We consider an output-arm loss model, in which we take a correlated pair consisting of p, a sample-interacting mid-IR line ("probe"), and r, a correlated optical comb line ("readout"). We then apply a sample transmission to the output of p from the cavity system

$$\begin{aligned} \hat{b}_p &= \sqrt{T_p}\hat{s}_{p,\text{out}} + \sqrt{1-T_p}\hat{v} \\ \hat{b}_r &= \hat{s}_{r,\text{out}}. \end{aligned} \tag{S6}$$

Here, $\hat{b}_p$ is derived from a beamsplitter interaction where $\hat{s}_{k,\text{out}}$ denotes the output annihilation operator for the cavity system and $\hat{v}$ denotes vacuum. The optical line is unperturbed – either it can be spectrally separated before interaction with the sample or it can pass through the sample if

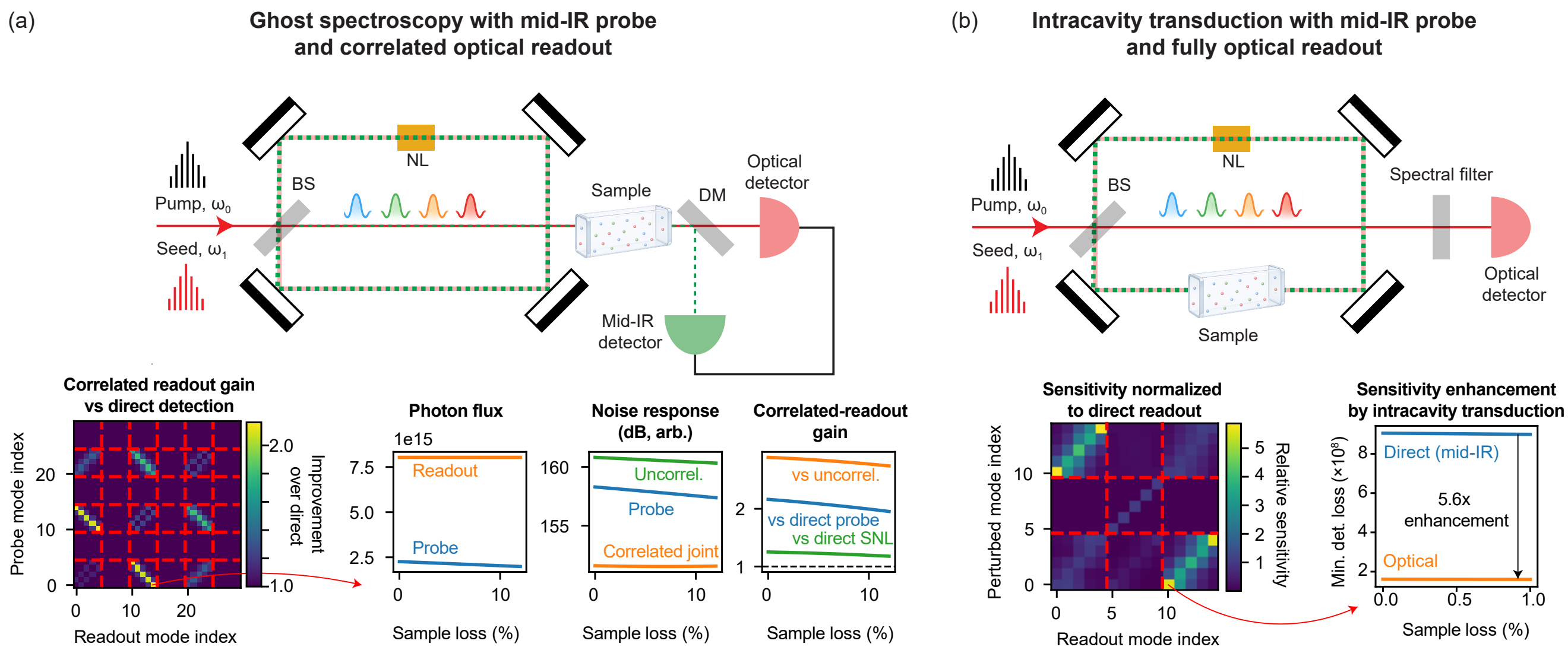

FIG. S2: **Ghost spectroscopy and intracavity transduction with quantum correlated frequency combs.** (a) Ghost spectroscopy setup where the mid-IR comb from the cavity system passes through a sample outside the cavity. Correlation-enhanced measurements allow improved sensitivity compared to direct mid-IR detection. Here, $I = 5$, $J = 5$, optical outcoupling loss is 25%, intrinsic loss for all modes is 1%, the pump power is 150 W, and the seed power is 0.01 W (the Gaussian spectral profile of the pump and seed remains the same as in the main text). (b) Intracavity transduction setup where the sample is placed inside the cavity and influences the nonlinear dynamics. Again through three wave mixing-mediated quantum correlations, it is possible to gain improved sensitivity in an optical readout channel that has never interacted with the sample. Here, $I = 2$, $J = 5$, the pump power is 50 W, and the seed power is 3000 W.

it is optically transparent. The goal is to use an optimized weighted joint observable

$$\delta\hat{I}_g = \delta\hat{n}_p - g\delta\hat{n}_r \tag{S7}$$

to achieve enhancement over direct detection using $\delta\hat{n}_p$. Denoting $\alpha = 1 - T_p$, the output loss channel performs the transformation $n_p \to (1-\alpha)n_p$ and the signal slope is given by

$$\frac{dS}{d\alpha} = \frac{dn_p}{d\alpha} = n_p|_{\alpha=0}. \tag{S8}$$

The variance of the joint observable is

$$\mathrm{Var}(\hat{I}_g) = \mathrm{Var}(\hat{n}_p) - 2g\mathrm{Cov}(\hat{n}_p, \hat{n}_r) + g^2\mathrm{Var}(\hat{n}_r) \tag{S9}$$

and the minimum detectable absorption is defined as noise over slope for observable $\mathcal{O}$

$$\Delta\alpha_{\min}|_{\mathcal{O}} = \frac{\sqrt{\mathrm{Var}(\hat{\mathcal{O}})}}{|dS/d\alpha|}, \tag{S10}$$

giving three baselines to compare:

$$\begin{aligned} \Delta\alpha_{\min}^{\text{direct}} &= \frac{\sqrt{\mathrm{Var}(\hat{n}_p)}}{n_p|_{\alpha=0}} && \text{Direct probe} \\ \Delta\alpha_{\min}^{\text{joint}} &= \frac{\sqrt{\mathrm{Var}(\hat{I}_g)}}{n_p|_{\alpha=0}} && \text{Joint correlated readout} \\ \Delta\alpha_{\min}^{\text{direct,SNL}} &= \frac{\sqrt{n_p}}{n_p|_{\alpha=0}} && \text{Direct probe, SNL.} \end{aligned} \tag{S11}$$

In Fig. S2a, we first show a heatmap indicating the improvement factor over direct detection for all pairs of probe and readout modes, $\Delta\alpha_{\min}^{\text{joint}}/\Delta\alpha_{\min}^{\text{direct}}$. We then focus on the specific case of a mid-IR probe and optical readout, demonstrating correlated-readout gains over twice the direct probe detection limit and over 25% below the shot noise limited direct probe detection limit. This enhancement is not limited to one comb line; we calculate enhancements over the SNL from 17% to 25% for all comb lines in the mid-IR comb for this example. We have found configurations in which the correlated-readout gain can reach 10 times above the direct probe detection limit, but these systems often feature excess single mode intensity noise that masks squeezing below the shot noise limit. These configurations may be of relevance when noisy (e.g., high power) lasers are necessary for an application, in which case the correlated detection offers a significant enhancement over direct detection, approaching or surpassing the shot noise limit. Sec. G G.1 considers an optimization of the gain factor over shot noise limited direct detection.

C.2. Intracavity transduction sensing

We next consider an example in which the observable can be based purely in a single optical readout channel, with no need for joint detection. The sample is placed inside the cavity and can influence the nonlinear dynamics. In this case, we show that a purely optical readout has higher intrinsic sensitivity than the mid-IR idler that has interacted with the sample. This arises because

of quantum correlations that link the mid-IR modes with optical modes. We add a perturbative intrinsic loss to comb line p ("probe") corresponding to

$$\begin{aligned}\dot{a}^T_{-k} &= \sum_{ij} \beta_{ijk} a^*_{ij} a_{i-1,j-k} - (\gamma_T + \mu_T + \delta_{kp}\mu_p) a^T_{-k} \\ \dot{a}_{ij} &= \sum_k \left[-\beta^*_{i+1,j+k,k} a^T_{-k} a_{i+1,j+k} + \beta_{ijk} a^{T*}_{-k} a_{i-1,j-k} \right] \\ &\quad - (\gamma_{ij} + \mu_{ij}) a_{ij} + \sqrt{2\gamma_{ij}} s_{ij}.\end{aligned} \tag{S12}$$

Similar to the remote loss sensing example above, we calculate the minimum detectable loss as

$$\delta\mu^{(r)}_{\min} = \frac{\sqrt{\mathrm{Var}(\hat{P}_r)}}{|\partial P_r / \partial \mu_p|}. \tag{S13}$$

Fig. S2b plots the relative intrinsic sensitivity for all possible pairs of probe (perturbed) and readout modes, $\delta\mu^{(r)}_{\min}/\delta\mu^{(p)}_{\min}$. We then focus on a specific mid-IR comb line as the probe, demonstrating 5.6x intrinsic sensitivity enhancement with a purely optical readout. Practically, this figure would likely be higher given the better detectors accessible at optical wavelengths.

D. COUPLED GENERALIZED NONLINEAR SCHRÖDINGER EQUATIONS

We simulate ultrafast pulse propagation through a medium phase-matched for cascaded difference and sum frequency generation via the following set of nonlinear Schrödinger equations [S2]:

$$\begin{aligned}\frac{\partial a_T(z,t)}{\partial z} - \hat{L}_T(t) a_T + \left(\frac{1}{v_T} - \frac{1}{v_{\mathrm{ref}}}\right)\frac{\partial a_T}{\partial t} &= \sum_{k\neq T} \beta^L_k(z) a^*_k a_{k-1} + i\Gamma^L_T \sum_k g_{kT} |a_k|^2 a_T \\ \frac{\partial a_n(z,t)}{\partial z} - \hat{L}_n(t) a_n + \left(\frac{1}{v_n} - \frac{1}{v_{\mathrm{ref}}}\right)\frac{\partial a_n}{\partial t} &= (\beta^L_n(z) a^*_T a_{n-1} - \beta^{L*}_n(z) a_T a_{n+1}) + i\Gamma^L_n \sum_k g_{kn} |a_k|^2 a_n,\end{aligned} \tag{S14}$$

where $g_{nn'} = \delta_{nn'} + 2(1-\delta_{nn'})$ (for co-polarized beams at different frequencies, which is the case for type-0/d_{33} QPM in PPLN, our baseline platform) and the dispersion operator is given by

$$\hat{L}_k(t) a_k = -\frac{\alpha_k}{2} a_k + \sum_{m\geq 2} \frac{i^{m+1}}{m!} D_{mk} \frac{\partial^m a_k}{\partial t^m}, \tag{S15}$$

where α_k denotes the loss per unit length and D_{mk} are the dispersion coefficients. The second-order nonlinear strengths are quantified by $\beta_k(z) = \beta e^{-i\Delta k_n z}$ where $\Delta k_n = k_{n-1} - k_n - k_T - K_{nn'}$ is the wavevector mismatch for each three-wave mixing interaction ($K_{nn'}$ denotes the grating vector). Unless otherwise specified, we will assume perfect phase matching between the central frequencies of nearest neighbor pulses ($\Delta k_n = 0$), as achieved by a composite nonlinear crystal (or multiple nonlinear crystals) quasi-phase matched for each three-wave mixing interaction.

The group-delay mismatch in each cascaded three-wave mixing interaction is represented by the term $(v_n^{-1} - v_{\text{ref}}^{-1})\frac{\partial a_n}{\partial t}$ where $v_n = d\omega_n/dk_n$ is the group velocity for the pulse at frequency ω_n. v_{ref} denotes the group velocity of the reference frame in which we work (usually chosen to be one of the pulses). This group-delay mismatch term is significant for ultrafast (femtosecond) pulses, where the walkoff during pulse propagation is comparable to the pulse width. Note that the single pulse case recovers the conventional GNLSE with $v_n = v_{\text{ref}}$.

For computational simplicity, we absorb the group velocity mismatch term (first order time derivative) into the dispersion operator

$$\hat{L}_k(t)a_k \to \left(\hat{L}_k(t) - \left(\frac{1}{v_k} - \frac{1}{v_{\text{ref}}}\right)\frac{\partial}{\partial t}\right)a_k \tag{S16}$$

and Fourier transform to

$$\tilde{a}'_k(z,\omega) = \tilde{a}_k(z,\omega)\exp\left(-\tilde{L}_k(\omega)z\right) \tag{S17}$$

so that

$$\frac{\partial \tilde{a}'_k}{\partial z} = \left(\frac{\partial \tilde{a}_k}{\partial z} - \tilde{L}_k(\omega)\tilde{a}_k\right)\exp\left(-\tilde{L}_k(\omega)z\right). \tag{S18}$$

Using any appropriate ODE solver, we can now evolve the fields from $\tilde{a}'_k(0,\omega)$ to $\tilde{a}'_k(z,\omega)$, simulating propagation through a length z in the nonlinear crystal.

E. ULTRAFAST MEAN-FIELD AND NOISE PROPAGATION MODEL

Our final model consists of a set of coupled pulsed fields propagating through a $\chi^{(2)}$ nonlinear crystal with additional Kerr-type self- and cross-phase modulation (following Eq. S18), followed

by cavity roundtrip loss and optional temporal resynchronization. The simulated fields are complex envelopes $a_j(t)$ for each pulse, defined in the time domain. For one crystal pass, the fields evolve under a split-step propagation consisting of linear dispersive propagation in frequency space and nonlinear coupling in time space. The nonlinear step includes both the cascaded three-wave-mixing interactions between neighboring subcomb fields and the idler, and an intensity-dependent $\chi^{(3)}$-like phase shift. After each pass, the fields are attenuated by the roundtrip transmission. The mean field is obtained by iterating this classical roundtrip map for the chosen number of roundtrips

$$a^{(r+1)} = \mathcal{R}\left[\mathcal{P}\left(a^{(r)}\right)\right], \tag{S19}$$

where $\mathcal{P}$ denotes one nonlinear crystal pass and $\mathcal{R}$ the roundtrip loss operator. Starting from the input pulse set $a^{(0)}$, this yields the output mean field a_{out}. The corresponding photon-normalized temporal amplitudes are then obtained by rescaling each field according to its carrier frequency and the simulation time step $a_j \to b_j$, so that $|b_j(t_n)|^2$ represents photon number per time bin.

Quantum noise is treated in the Gaussian linearization (small-fluctuation) approximation about the classical mean-field trajectory. Vacuum fluctuations are introduced at the input and, importantly, fresh vacuum fluctuations are also injected at every lossy roundtrip channel through an explicit beamsplitter model, noting that the Fano factor after linear transmission α behaves as $F_{\text{in}} \to F_{\text{out}} = \alpha F_{\text{in}} + (1-\alpha)$. The output observables are expanded to first order in these input vacuum quadratures, and the Jacobian J of the output observable with respect to the input quadratures is computed by automatic differentiation. The output covariance matrix is then

$$V_{\text{out}} = J V_{\text{vac}} J^T, \tag{S20}$$

with $V_{\text{vac}} = \frac{1}{4} I$ for vacuum input in the real-quadrature convention used here. This procedure ensures that passive attenuation alone does not produce artificial squeezing: a shot-noise-limited input remains shot-noise limited in the absence of nonlinear gain.

Two classes of observables are evaluated. In the mode-projection analysis, the photon-normalized output field is projected onto chosen temporal or spectral observer modes, yielding mean quadratures and their covariance matrix. In the direct-detection analysis, photon number is integrated over chosen temporal or spectral bins, yielding mean binned photon numbers and their covariance matrix. From these moments, we compute noise-reduction factors for gain-balanced intensity-

difference measurements normalized to the shot noise limit,

$$\mathrm{NRF}|_{\mathrm{SNL}} = \frac{\mathrm{Var}(N_s - gN_i)}{\langle N_s + g^2 N_i \rangle}, \tag{S21}$$

where g may be chosen as unity, mean-balancing, or the covariance-optimal gain. Thus, our simulation predicts both the classical output pulse shapes and the quantum noise properties of experimentally relevant integrated observables such as whole-pulse or binned photon-flux correlations.

F. COVARIANCE MATRIX OPTIMIZATION PROCEDURE

F.1. Discrete entanglement model

We optimize a bounded set of cavity and drive parameters in the discrete mean-field plus linearized-noise model. For a system with I optical subcombs and J lines per comb, the parameter vector contains:

- Optical outcoupling roundtrip loss for each optical subcomb, $\{L_{s,i}^{\mathrm{out}}\}_{i=1}^{I}$
- Idler outcoupling loss, L_T^{out}
- Intrinsic roundtrip loss for each optical subcomb, $\{L_{s,i}^{\mathrm{int}}\}_{i=1}^{I}$
- Intrinsic idler roundtrip loss, L_T^{int}
- Total pump- and seed-power fractions relative to the baseline budgets
- The spectral distribution of pump and seed power across the J driven lines.

The pump and seed line-by-line powers are parameterized by unconstrained logits and mapped to nonnegative profiles with a softmax, so that the total pump and seed powers remain bounded by fixed budgets:

$$\begin{aligned} P_j^{(\mathrm{pump})} &= P_{\max}^{(\mathrm{pump})} \frac{e^{u_j}}{\sum_k e^{u_k}} \\ P_j^{(\mathrm{seed})} &= P_{\max}^{(\mathrm{seed})} \frac{e^{v_j}}{\sum_k e^{v_k}}, \end{aligned} \tag{S22}$$

where $P_{\max}$ is taken from the baseline configuration, so the optimizer reallocates power spectrally and can reduce the total used power, but does not exceed the reference pump or seed budget.

Each trial parameter set is evaluated by:

1. Building the corresponding discrete cavity model
2. Solving the nonlinear steady state
3. Computing the linearized output noise and covariance matrix
4. Extracting a scalar figure of merit corresponding to the specified objective function

To prevent the optimizer from selecting dynamically unstable or non-converged operating points, we add a penalty

$$\mathcal{L}_{\text{tot}} = \mathcal{L}_{\text{metric}} + \mathcal{P}, \tag{S23}$$

where the penalty $\mathcal{P}$ increases when the steady state solver fails to converge to the required tolerance or the linearized drift matrix has a positive largest real eigenvalue, indicating instability. Thus, the reported optimum is the best point that jointly improves the target metric while remaining near a valid steady state and a stable linearized operating point.

The starting point is the baseline configuration together with the default Gaussian pump and seed line profiles. Bounds are imposed on all optimized quantities. A notable constraint is that intrinsic losses are only allowed to increase relative to their initial values, not decrease below them. This treats intrinsic loss as a fixed physical floor, while allowing the optimizer to vary coupling losses and drive distributions as practical design knobs.

The outer optimization is performed with bounded Powell search, using:

- The baseline parameter vector as one start,
- Additional uniformly random bounded starts,
- The best objective value across all runs as the reported result.

This choice was used because the objective is nonconvex, can be nonsmooth when the best pair is selected by scanning, and includes embedded nonlinear solves.

F.2. Ultrafast pulse model

In Fig. 5 of the main text, we optimize the pump and seed pulse shapes in the differentiable time-domain quantum sensitivity analysis (QSA) model to target a desired correlation or squeezing pattern. Rather than optimizing every time sample independently, the pump and seed fields are expanded in low-dimensional orthonormal Hermite-Gaussian bases:

$$
\begin{aligned}
A_{\text{pump}}(t) &= \sum_{n=0}^{N_p-1} c_n^{(p)} \phi_n^{(p)}(t) \\
A_{\text{seed}}(t) &= \sum_{n=0}^{N_s-1} c_n^{(s)} \phi_n^{(s)}(t).
\end{aligned}
\tag{S24}
$$

The basis centers and widths are taken from the initial pulse centroids and rms widths unless specified otherwise. The complex basis coefficients are the main optimization variables. Additional control variables are:

- Spectral chirp coefficients for user-selected polynomial orders
- Relative seed delay
- Intrinsic roundtrip losses for selected fields.

For each trial set of coefficients, the code:

- Reconstructs the pump and seed temporal envelopes from the basis,
- Optionally applies spectral phase chirp in the frequency domain,
- Optionally applies a seed time delay,
- Renormalizes the pump to its initial pulse energy,
- Renormalizes the seed to its initial pulse energy or clamps it to a prescribed maximum energy.

This keeps the optimization focused on pulse shape rather than trivially increasing energy.

The optimized target is a matrix of pairwise noise metrics between output channels. Depending on configuration, the observable can be built from:

- Grouped intensity bins, or
- Grouped observer/supermode projections,

in either the time or frequency domain. The resulting profile matrix is typically the shot-noise-normalized noise-reduction factor matrix, although normalization to the uncorrelated reference can also be used.

The main loss is a weighted mean-squared mismatch between the trial profile matrix and a target matrix:

$$\mathcal{L}_{\text{target}} = \frac{\sum_{mn} w_{mn} \left(M_{mn} - M_{mn}^{\text{target}}\right)^2}{\sum_{mn} w_{mn}}. \tag{S25}$$

Diagonal entries are excluded, and undefined target entries are masked out. The total loss is

$$\mathcal{L} = \mathcal{L}_{\text{target}} + \lambda_{\text{bright}}\mathcal{L}_{\text{bright}} + \lambda_{\text{anchor}}\mathcal{L}_{\text{anchor}} + \lambda_{\text{smooth}}\mathcal{L}_{\text{smooth}} + \lambda_{\text{chirp}}\mathcal{L}_{\text{chirp}} + \lambda_{\text{loss}}\mathcal{L}_{\text{loss}}. \tag{S26}$$

The additional terms are:

- Brightness penalty: penalizes solutions whose mean mode photon numbers fall below a minimum threshold,
- Shape-anchor penalty: penalizes large deviations from the initial pump and seed pulses,
- Smoothness penalty: penalizes large second differences of the temporal envelopes,
- Chirp-anchor penalty: penalizes large chirp coefficients,
- Loss-anchor penalty: penalizes large deviations of optimized intrinsic losses from the initial values.

These regularizers suppress unphysical or overly distorted pulse shapes and bias the search toward experimentally plausible solutions.

We generally employ a "full Powell" optimization strategy, where Powell directly optimizes the pulse-basis coefficients together with chirp, delay, and intrinsic losses in one bounded derivative-free search.

G. OTHER OPTIMIZATION EXAMPLES

G.1. Remote loss sensing optimization

Fig. S3a/b presents an optimization where the objective is to maximize the gain over shot noise limited direct detection. As with the other optimizations considered in this paper, a global optimum cannot be guaranteed. However, we observe an optimized gain approaching twice the shot noise limited sensitivity.

G.2. Cluster state optimization

Fig. S3c presents an optimization where the objective is to minimize the maximum nullifier variance for three different star graphs. The pump and seed profile were included as optimizable parameters, but these particular optimizations primarily changed the loss profile.

H. DESIGN CONSIDERATIONS AND ESTIMATION OF EXPERIMENTAL PARAMETERS

We believe that one of the most realistic near-term route for realizing the physics described in the paper is a periodically poled lithium niobate (PPLN)-based cascaded three-wave-mixing platform using either sequentially phase-matched crystals or a multi-period/apodized QPM structure. Multi-channel QPM in lithium niobate waveguides and cascaded DFG, OPG, and OPO processes have direct experimental precedent [S3–S7]. In the longer term, the preferred platform is dispersion-engineered thin-film lithium niobate waveguides/resonators, where millimeter-scale periodically poled devices with micron-scale mode areas, effective nonlinear coefficients d_{eff} of order 10–30 pm/V, and low propagation loss have already enabled efficient $\chi^{(2)}$ frequency conversion, broadband OPA/DFG, and visible-to-mid-IR comb generation [S8–S13].

In this manuscript, we focus on a free-space cavity platform using PPLN. While phase matching for a far-IR/terahertz idler is possible with bulk or singly-poled nonlinear crystals [S14], terahertz carries its own challenges, particularly with respect to loss; however, we note that single-frequency terahertz-mediated cascading has already been demonstrated in singly-poled PPLN (see [S15] and related works). Instead, in this work, we focus on a mid-IR idler. We propose two possible cavity

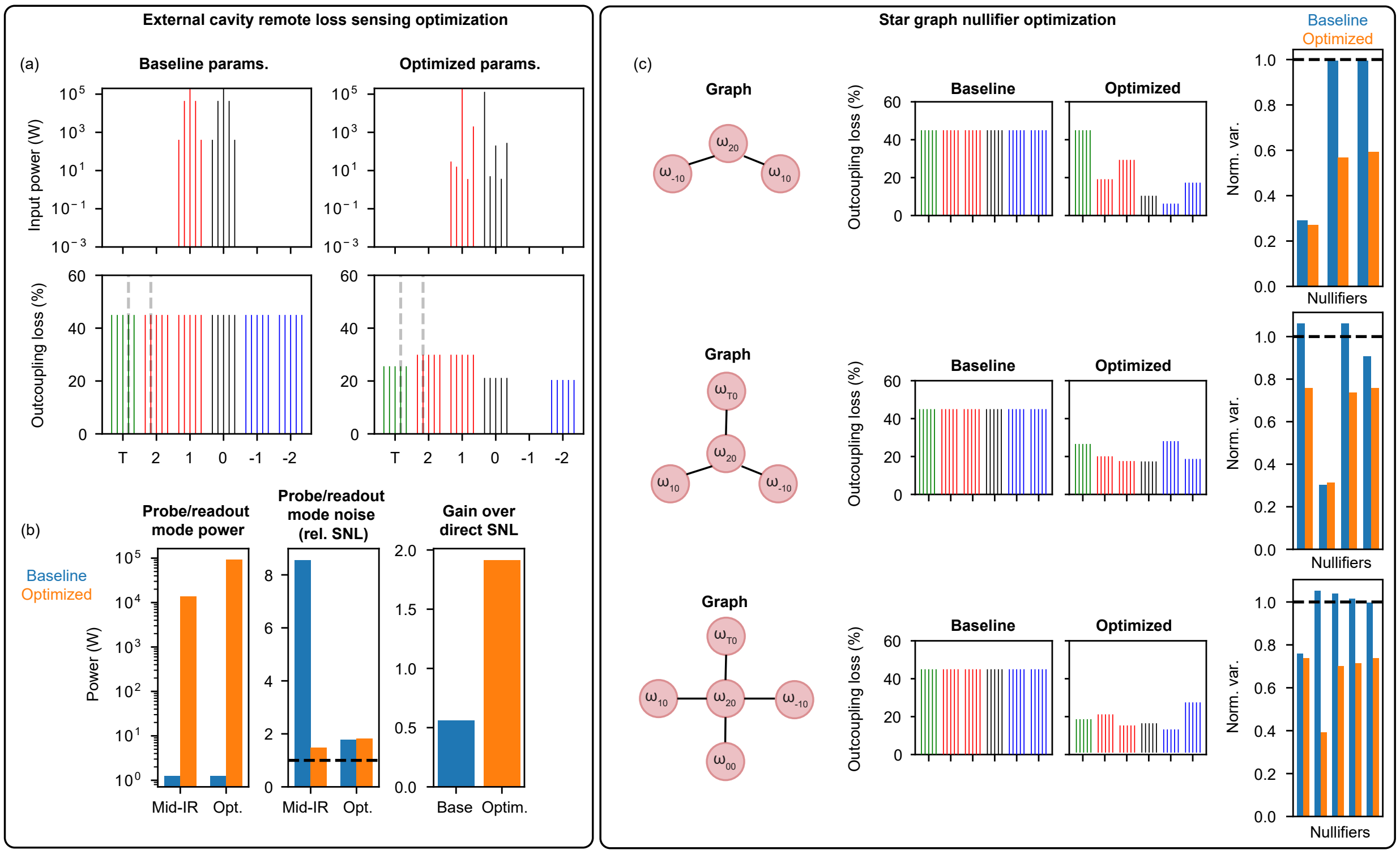

FIG. S3: **Remote loss sensing and cluster state nullifier optimization.** (a) For the remote loss sensing application: optimization over the pump/seed comb profile (constrained to not exceeding the initial pump/seed power budget) and outcoupling loss for the combs. (b) Baseline and optimized power and intensity noise in the mid-IR probe and optical readout modes, alongside the gain over shot noise limited direct detection. (c) Cluster state nullifier variance optimization for three different star graphs. Baseline parameters: seed and pump power 100 kW, intrinsic loss 5% for all modes.

geometries:

1. Single cavity in which the optical and mid-IR modes are resonant, with appropriate dispersion compensation optics. The cavity mirrors can be metallic ($<5\%$ loss for optical and mid-IR) or dual-band coated in the optical and mid-IR to minimize intrinsic losses further.

2. Dual-cavity setup in which the mid-IR idler is resonant in a separate cavity that is coupled to the optical cavity through dichroic mirrors. This eliminates the need for dual-band coatings on the cavity optics. For the optical cavity mirrors, a typical broadband dielectric coating can easily cover 5-7 combs through judicious choice of the pump and seed wavelengths. Gold-coated or narrowband-coated mirrors in the mid-IR cavity similarly offer low loss for the idler.

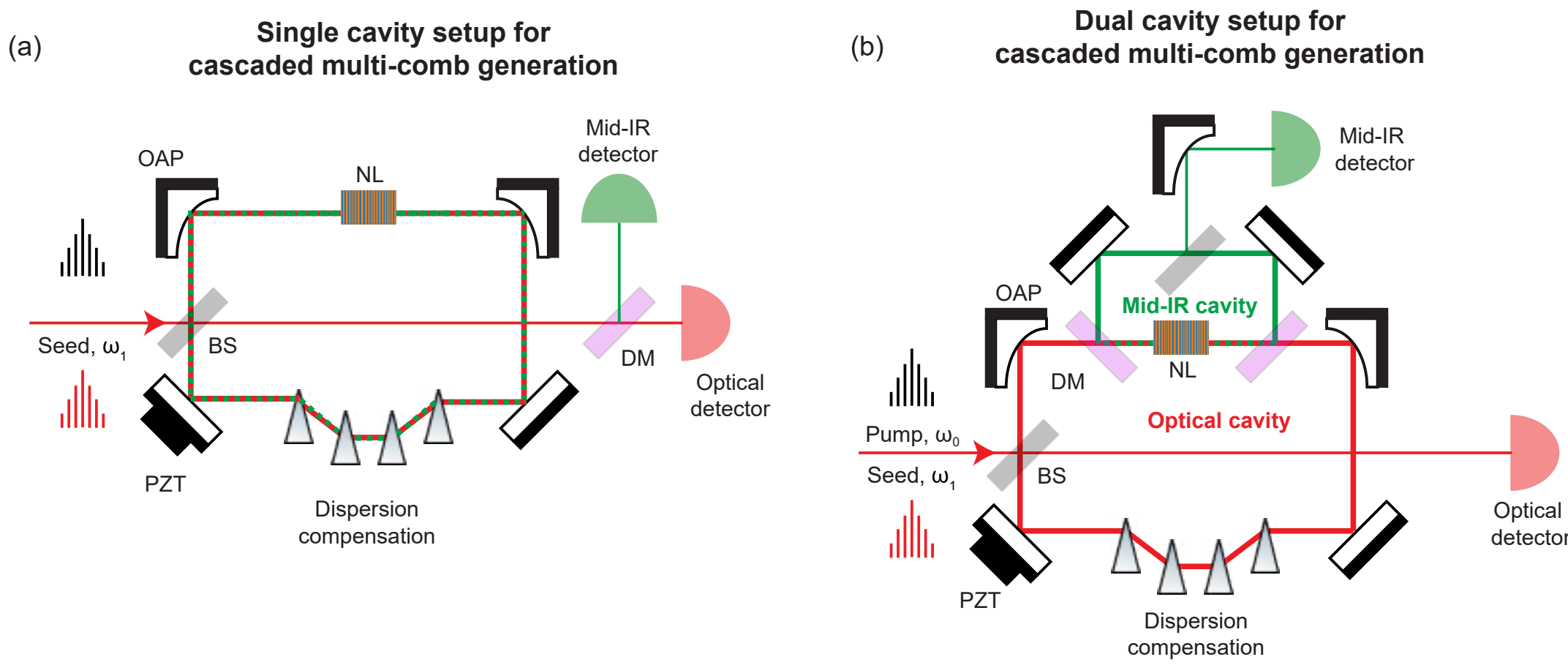

FIG. S4: **Schematic implementations of mid-IR-mediated cascaded comb generation.** (a) Single cavity setup, where the mid-IR idler and optical combs are resonant in a single cavity. (b) Dual cavity setup, where the mid-IR idler comb is resonant in a separate cavity from the optical combs. PZT: piezo-electric transducer for resonance tuning, BS: beamsplitter, DM: dichroic mirror, NL: nonlinear crystal (PPLN), OAP: off-axis parabolic mirror for mode matching.

H.1. Second order nonlinearity and phase matching

In this section, we provide an estimation of typical nonlinear strengths β in a free space multimode cavity. As derived from first-order perturbation theory [S16],

$$\beta_{ijk} = 2d_{\text{eff}}\sqrt{\frac{\hbar\omega_{ij}\omega_{i-1,j-k}\omega^T_{-k}}{\epsilon_0}}\frac{\int_{\text{NL}} dV\, E_{ij}E^*_{i-1,j-k}E^{T*}_{-k}}{\sqrt{\int_{\text{cav}} dV\,(\epsilon_r|E|^2)_{ij}}\sqrt{\int_{\text{cav}} dV\,(\epsilon_r|E|^2)_{i-1,j-k}}\sqrt{\int_{\text{cav}} dV\,(\epsilon_r|E|^2)^T_{-k}}}, \tag{S27}$$

where $\int_{\text{NL}}$ denotes integration over the volume of the nonlinear crystal and $\int_{\text{cav}}$ denotes integration over the entire cavity. For concreteness, we consider $LiNbO_3$, for which $d_{\text{eff}} \sim \mathcal{O}(20)$ pm/V [S17]. Then, with frequencies $\omega_{00} = 2\pi \cdot 645$ THz, $\omega_{10} = 2\pi \cdot 571$ THz, and $\omega^T_0 = 2\pi \cdot 73.7$ THz, we have refractive indices $n_{00,10} = \sqrt{\epsilon_r}|_{00,10} \approx 2.35$ and $n^T_0 = \sqrt{\epsilon_r}|^T_0 \approx 2.10$. We assume the electric field profiles $E_{00,10}, E^T_0$ are uniform over a cross-section $A \approx 1 \times 10^{-6}$ m^2 and that the crystal length is $L_{\text{NL}} \approx 10$ mm while the cavity length is $L_{\text{cav}} \approx 10$ cm. Then, we have for example

$$\begin{aligned}\beta_{100} &\sim \frac{2d_{\text{eff}}}{n_{00}n_{10}n^T_0}\sqrt{\frac{\hbar\omega_{00}\omega_{10}\omega^T_0}{\epsilon_0}}\frac{AL_{\text{NL}}}{(AL_{\text{cav}})^{3/2}} \\ &\approx 309\ \text{s}^{-1},\end{aligned} \tag{S28}$$

matching the order of magnitude for values of β_{ijk} considered in the main text, $\beta_0 = \beta_{100}/\sqrt{\hbar\omega_{00}\omega_{10}\omega_0^T} \sim 10^{-4}$ J$^{-1/2}$. This value changes depending on the cavity geometry and nonlinear material.

In addition to the nonlinear strength, phase matching constraints dictate which modes engage efficiently in three-wave mixing processes. As discussed in the main text, because multiple highly nondegenerate three-wave mixing processes are taking place in the multi-comb system, separate crystals or a single crystal with multiple poling periods are necessary to guarantee phase matching between each pair of neighboring subcombs. We assume perfect phase matching between the center frequencies $\omega_{i,0}, \omega_{i\pm1,0}$ of neighboring combs. The extent to which comb lines $j \neq 0$ is determined by the dispersion of the nonlinear crystal and its length, which together set the phase matching bandwidth.

Engineered multi-process quasi-phase matched (QPM) devices can be modeled through the spatial nonlinear pattern

$$\eta_{iji'j'} \propto \int_0^L d(z) e^{i\Delta k_{iji'j'} z} dz, \tag{S29}$$

where the nonlinear coefficient phase poling profile $d(z)$ will have multiple Fourier components or section-dependent phase for multi-period/phase-modulated gratings [S4, S7]. We assume a simplified model

$$\eta_{iji'j'} \propto e^{i\Delta k_{iji'j'} L/2} \mathrm{sinc}(\Delta k_{iji'j'} L/2) \tag{S30}$$

where $\Delta k_{iji'j'} = k_{ij} - k_{i'j'} - k^T_{j-j'} - K_{ii'}$ is the wavevector mismatch ($K_{ii'}$ is the grating vector). The crystal is designed with grating vectors

$$K_{ii'} = \begin{cases} k_{i0} - k_{i'0} - k_0^T & \text{if } i' - i = 1 \\ 0 & \text{otherwise.} \end{cases} \tag{S31}$$

We find that this simplified model shows adequate agreement with a more complex apodized model following Eq. S29 that incorporates Fourier weights for each QPM channel and ripple/crosstalk.

We also perturbed the designed QPM periods away from their ideal values and recomputed the full mean-field and noise models. As expected, shorter crystals are more robust to poling period

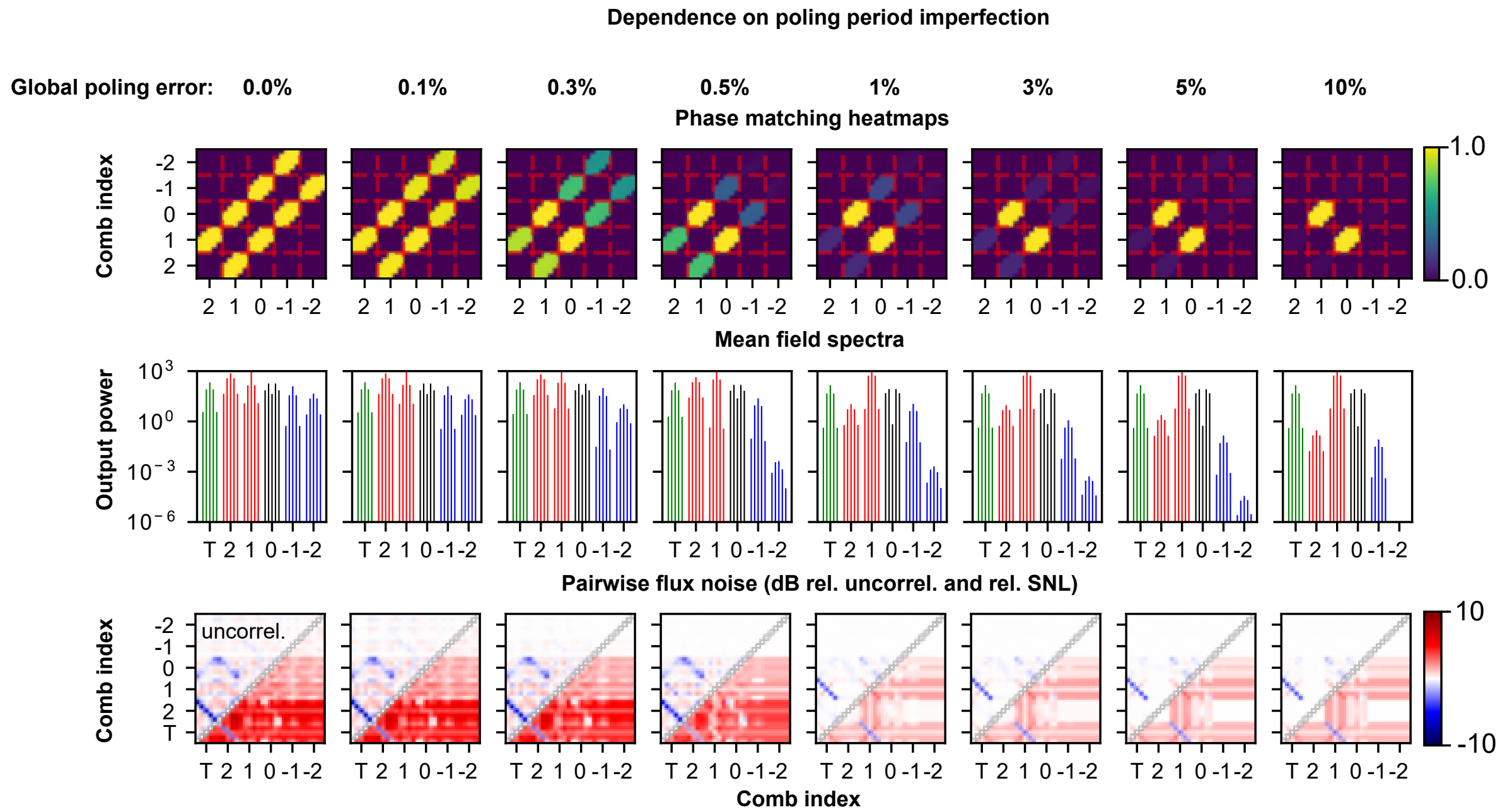

FIG. S5: **Effects of poling period errors.** Phase matching heatmaps, mean field spectra, and pairwise flux noise corresponding to different levels of global poling error ϵ applied to a 1 mm PPLN crystal, where the poling periods are $\Lambda_{\text{actual}} = \Lambda_{\text{ideal}}(1+\epsilon)$.

imperfections. In Fig. S5, we study the dependence of the mean field and two-mode noise on a global shift to all periods in the multi-poled structure. A 1 mm long crystal is used with ideal poling periods 12.2, 8.60, 6.96, and 5.34 micron.

H.2. Intracomb spacing

In Fig. S6, we show the dependence of the quantum correlations between different frequency modes on the comb spacing ω_m, which directly sets which modes will fall within the nonlinear crystal's phase matching bandwidth and therefore interact strongly with each other. The phase matching heatmaps corresponding to different ω_m are shown in the lower triangles of Fig. S6(a1,a2). The upper triangles show the two-mode intensity difference noise, expressed in dB relative to the uncorrelated noise. At small comb spacings < 10 GHz, the phase-matching coefficient is approximately constant between all pairs of modes in adjacent combs, enabling well-defined nonlinear coupling pathways and resulting in correlations between specific mode pairs.

As ω_m is increased to ~ 100 GHz, the relative detunings between interacting modes are reshuffled, leading to increasingly non-uniform phase matching and distribution of energy in the non-

linear system. Correspondingly, correlations become distributed across a larger number of mode pairs. In this regime, quantum noise is shared among many interacting modes, reflecting a transition from localized pairwise correlation to a delocalized multimode correlation landscape. The strength of correlations in this delocalized landscape is sensitive to ω_m (e.g., comparing $\omega_m = 10$ GHz and $\omega_m = 100$ GHz), which essentially governs which nonlinear interactions shape the energy flow in the multimode system. This in turn governs the interference between correlations coming from individual three-wave mixing processes (i.e. individual upconversion and downconversion processes). This suggests the potential of using dispersion-mediated control of the nonlinear coefficient to shape the covariance matrix of frequency-multimode systems.

H.3. Pump strengths and noise

Nanosecond pulsed lasers can easily reach 1 MW peak powers under Q switched operation [S18], while microsecond pulsed sources can enable quasi-CW operation at the level of kW or tens of kW. With a cavity designed to be centimeter scale ($\mathcal{O}(1)$ ns round-trip time), quasi-continuous wave (CW) operation is therefore possible, as considered in the main text. These laser sources typically have linewidths of tens of GHz, but injection-seeded versions of these lasers achieve linewidths in the MHz regime, allowing frequency combs to be generated from electro-optic modulation.

Ultrafast lasers provide a distinct regime for multi-comb operation. Mode-locked ultrafast lasers naturally produce frequency combs [S19]. For our simulations with picosecond pulses, we adopt pulse parameters typical of commercially available OPO/OPA systems.

We investigate the effect of injected pump intensity noise on multimode quantum correlations by evaluating the two mode intensity difference noise as a function of the pump Fano factor F_{in}. Excess noise is injected exclusively at the pump port. Results are presented for pump and signal seed powers $|s_{0,j}|^2 = 10$ W and $|s_{1,j}|^2 = 100$ mW, respectively. Here the intensity difference noise is normalized to the uncorrelated reference, defined as the noise expected if the two modes retained their individual intensity noise but exhibited zero cross-correlation.

Within our model, pump noise enters solely through a rescaling of the external Langevin correlators associated with the pumped optical modes by the factor F_{in}, while the linearized fluctuation dynamics encoded in the fluctuation matrix $\mathbf{M}(\Omega)$ remain unchanged. Consequently, the inverse of the fluctuation matrix $\mathbf{M}^{-1}(\Omega)$, which governs multimode mixing and noise transfer, is inde-

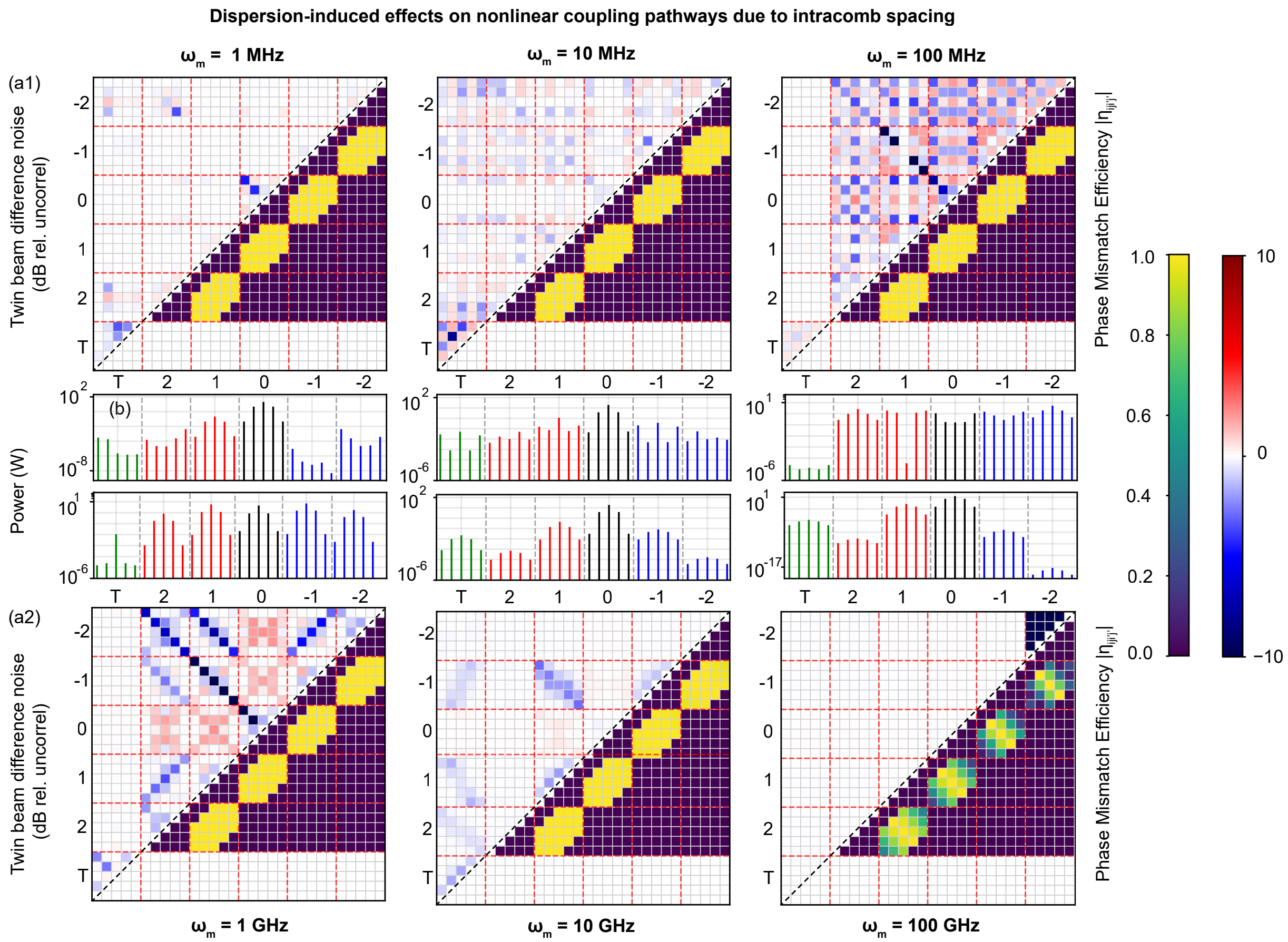

FIG. S6: **Effect of comb spacing on quantum correlations.** (a1,a2) As ω_m is varied, we plot the corresponding two-mode intensity difference noise (in dB relative to uncorrelated two-mode noise) together with dispersion-induced phase matching efficiency between individual modes $|\eta_{iji'j'}|$, illustrating how changes in comb spacing modify the effective phase matching and the resulting multimode correlations. (b) Associated steady-state mean fields for each value of ω_m. Here, the pump and seed powers are $|s_{0,j}|^2 = 10$ W and $|s_{1,j}|^2 = 100$ mW, respectively. The nonlinear coupling coefficient is $\beta_0 = 10^{-4}\ \mathrm{J}^{-1/2}$, the optical comb modes have quality factor $Q_s = 6.7\times10^7$, and the idler (THz) comb has quality factor $Q_T = 1.46\times10^6$. The pump wavelength is $\lambda_{0,j} = 465$ nm, and the idler wavelength is $\lambda_{T,j} = 4.07\ \mu$m ($\omega_T = 2\pi \times 73.7$ THz).

pendent of F. For two modes, the intensity difference variance is written as

$$V_{kk'} = \langle \delta\hat{n}_k^\dagger \delta\hat{n}_k \rangle + \langle \delta\hat{n}_{k'}^\dagger \delta\hat{n}_{k'} \rangle - 2\,\mathrm{Re}\langle \delta\hat{n}_k^\dagger \delta\hat{n}_{k'} \rangle, \tag{S32}$$

where $\langle \delta\hat{n}_k^\dagger \delta\hat{n}_k \rangle$ denotes the single-mode output noise/variance (in terms of the number fluctuation operator $\delta\hat{n}_k$) and $\langle \delta\hat{n}_k^\dagger \delta\hat{n}_{k'} \rangle$ is the cross correlator (covariance) between the two modes of interest.

Normalization to the uncorrelated reference yields

$$R_{\mathrm{unc},kk'} = \frac{V_{kk'}}{\langle \delta\hat{n}_k^\dagger \delta\hat{n}_k \rangle + \langle \delta\hat{n}_{k'}^\dagger \delta\hat{n}_{k'} \rangle} = 1 - \frac{2\,\mathrm{Re}\langle \delta\hat{n}_k^\dagger \delta\hat{n}_{k'} \rangle}{\langle \delta\hat{n}_k^\dagger \delta\hat{n}_k \rangle + \langle \delta\hat{n}_{k'}^\dagger \delta\hat{n}_{k'} \rangle}, \tag{S33}$$

which depends only on the ratio of the covariance to the total single mode noise.

Because both variance and covariance are linear functionals of the same Langevin noise sources weighted by the same susceptibility matrix $\mathbf{M}^{-1}(\Omega)$, increasing the pump Fano factor predominantly rescales these quantities proportionally. As a result, $R_{\mathrm{unc},kk'}$ remains nearly invariant over a broad range of F_{in}, even though the absolute noise level increases substantially. This behavior contrasts with representations normalized to the shot-noise limit, where the normalization retains the absolute noise baseline set by the output mean fields and therefore exhibits a pronounced degradation as pump noise increases.

The weak dependence of the uncorrelated normalized noise on F_{in} observed in both the strongly and weakly seeded regimes thus indicates that pump noise primarily acts as a common mode noise source, amplifying the overall noise floor without significantly altering the normalized inter-mode correlation structure.

H.4. Losses

The distribution of multimode quantum correlations in the cascaded nonlinear cavity is governed by the competition between coherent intermode energy exchange and comb-/mode-dependent dissipation. In Fig. S8a/b, we investigate the dependence of the mean field spectrum and single- and two-mode noise on intrinsic loss. Squeezing, as expected, decreases as intrinsic loss is increased, but significant squeezing is still observed even for large amounts of intrinsic loss, as shown in Fig. S8b. The squeezing also exists even though all frequency modes have individual intensity noise above the shot noise limit (Fig. S8a).

For the PPLN-based free space cavity-type system considered in the main text, intrinsic coupling losses arise from the finite reflectivity of the cavity mirrors and the absorption of PPLN (the wavelength of the mid-infrared idler can be chosen to lie in one of air's transparency windows).If metallic mirrors are used, commercially available options reach $> 98\%$ reflectivity in both the mid-IR and optical wavelengths we consider. In both of the aforementioned cavity geometries, a

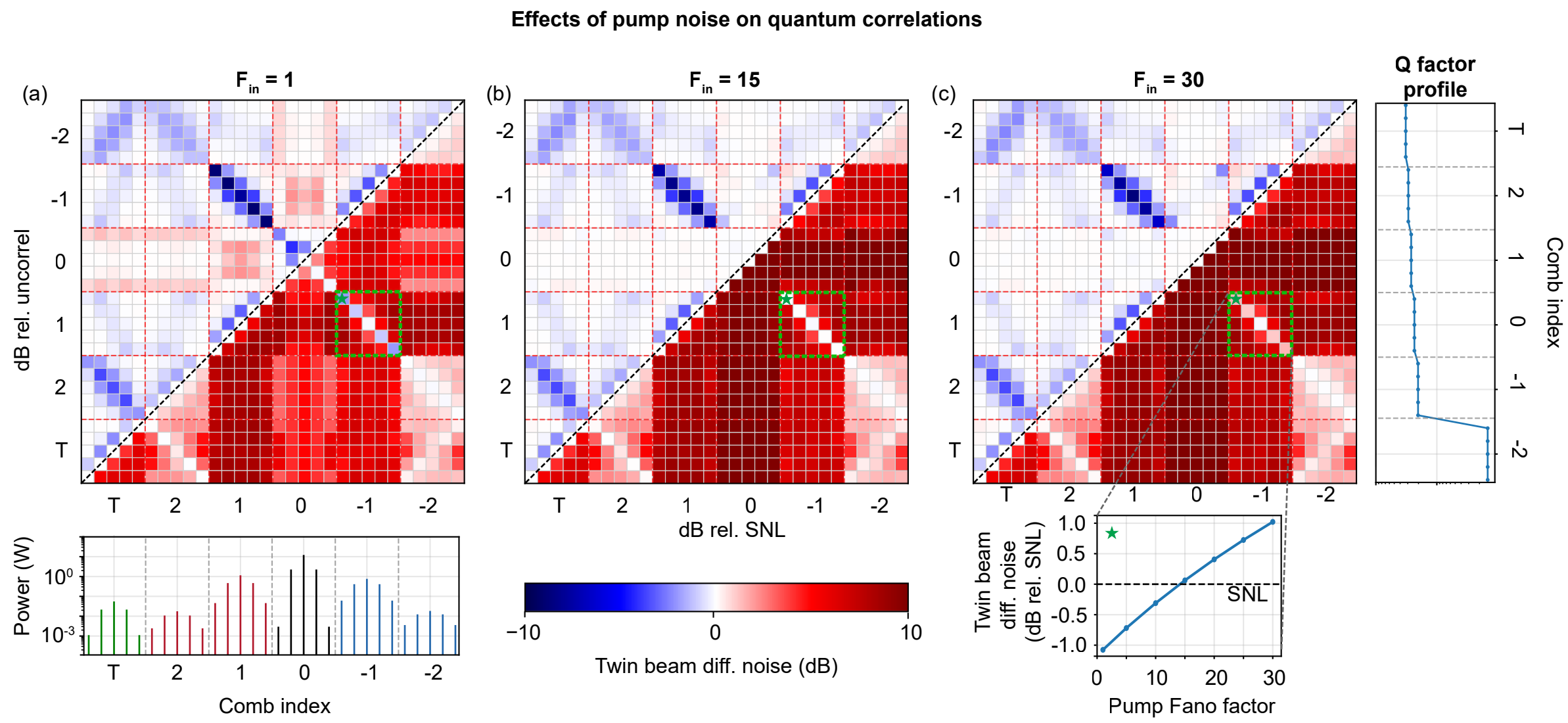

FIG. S7: **Effect of pump noise on multimode quantum correlations.** Two-mode intensity difference noise, expressed in dB relative to the uncorrelated and shot-noise limit (SNL) , as a function of injected pump noise characterized by the pump Fano factor F_{in}. Excess noise is injected at the pump port. The pump and signal seed powers are $|s_{0,j}|^2 = 10$ W and $|s_{1,j}|^2 = 100$ mW, respectively. As the pump noise increases, the quantum correlations remain strong, but the overall noise of the two-mode states increases with respect to the shot noise limit, as shown at far right for the frequency mode pair $\{\omega_{1,-2}, \omega_{-1,2}\}$ (indicated by a green star within the green bounding box), indicating reduced noise suppression between correlated mode pairs. (a), (b), and (c) demonstrate this trend for all pairs of modes for three different values of F_{in}.

dual-band AR coating on the nonlinear crystal covering the idler and optical subcombs would allow minimal intrinsic losses. However, we recognize that this can be demanding from a fabrication point of view.

Regarding absorption, lithium niobate remains a strong candidate owing to its large transparency window stretching from around 300 nm to 5 μm. The absorption coefficient of lithium niobate over this region has been characterized previously [S20], demonstrating absorption coefficients < 0.01 cm^{-1} from 500 nm to roughly 3 micron (with the exception of a few sharp peaks between 2 and 3 micron that can easily be avoided). This corresponds to $< 1\%$ loss for a 10 mm crystal. The highest absorption is around 4 micron (0.1 cm^{-1}), corresponding to 1% loss for a 1 mm crystal (10% for a 10 mm crystal). Shorter crystal lengths are also preferable for minimizing sensitivity to fabrication imperfections in the poling periods (see the previous section "Second order nonlinearity and phase matching").

Heuristically speaking, the strength of two-mode squeezing increases as $\mu/(\mu + \gamma)$ decreases,

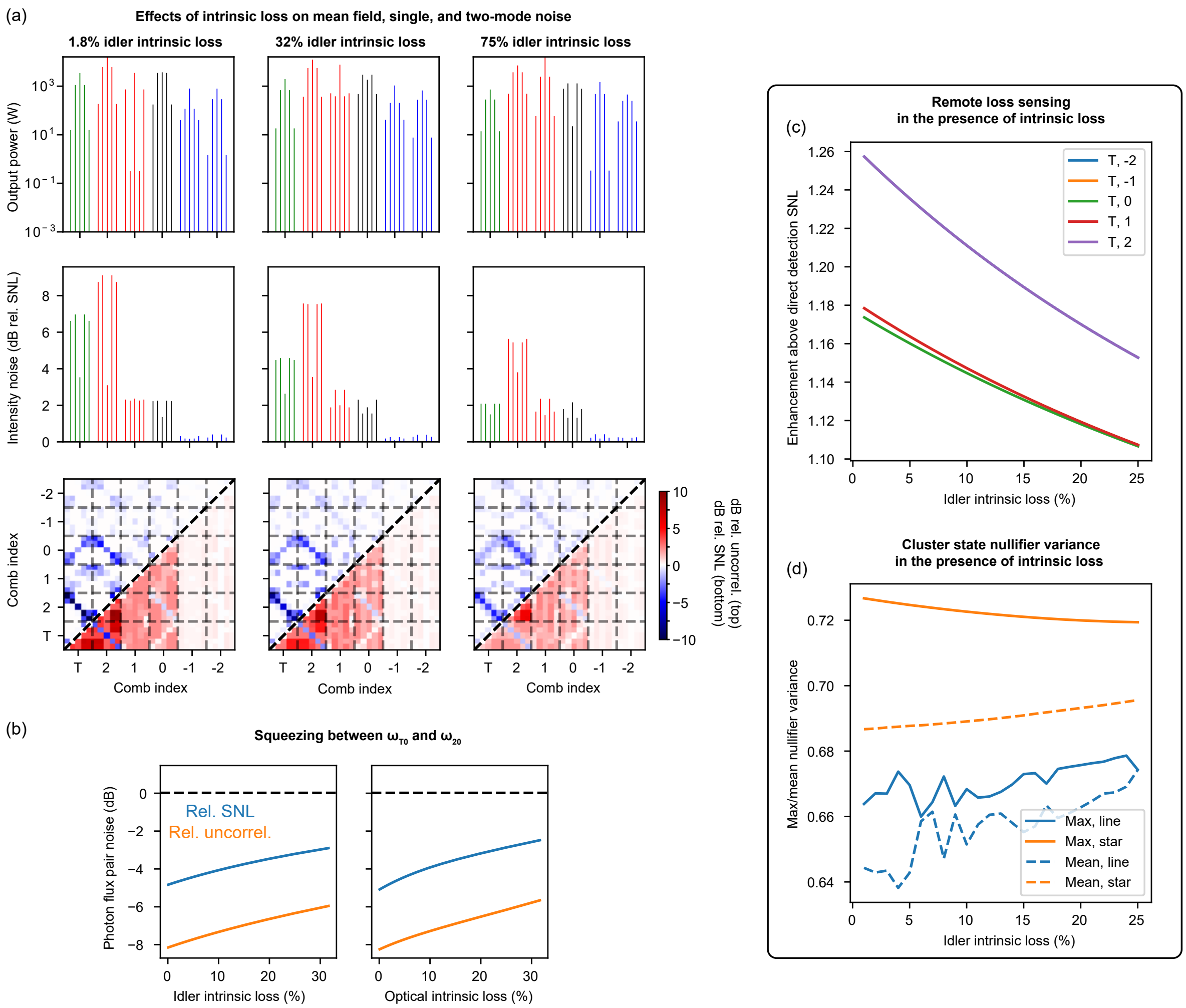

FIG. S8: **Effect of intrinsic quality factor of idler comb on multimode quantum correlations.** (a) Mean field, single mode intensity noise, and photon flux pair noise for varying levels of roundtrip idler intrinsic loss. (b) Evolution of photon flux pair noise between ω_{T0} (idler comb) and ω_{20} (optical comb) as both idler intrinsic loss (left) and optical intrinsic loss (right) are varied. Squeezing is maintained even for intrinsic loss levels exceeding 30%. System parameters: $I = J = 5$, 45% outcoupling loss for all modes, 5% intrinsic loss for modes whose intrinsic loss is not varied, and pump/seed power 10 kW. (c) Enhancement factor of correlated joint detection in the remote loss sensing example considered in the main text over direct detection with a shot noise limited source as a function of idler intrinsic loss. Enhancement factors for each of the comb lines in the mid-IR idler comb are presented. (d) Maximum and mean nullifier variances for the star and line graphs considered in the main text as a function of idler intrinsic loss. In both plots, the optical intrinsic loss is 5% and outcoupling loss for optical and idler modes is 50%. In (c), $I = 5$ and the maximum seed and pump powers are 1 W and 4.8 kW respectively. In (d), $I = 3$ and the maximum seed and pump powers are 10 kW and 50 kW respectively.

where μ denotes intrinsic loss and γ denotes outcoupling loss. This has been corroborated by studies of quantum noise in optical parametric oscillators (OPOs) [S21]. Therefore, it is possible to maintain significant squeezing and entanglement even in the presence of non-negligible

intrinsic loss if the outcoupling loss is also increased. This of course requires larger pumping strengths/stronger nonlinearity. Below, we evaluate the results in the manuscript for varying levels of intrinsic loss, and we observe multipartite entanglement and sub-SNL behavior even with intrinsic loss levels in excess of 20%. For reference, if the cavity was only optimized for the optical subcombs, we may anticipate around 15% intrinsic loss for the idler, dominated by Fresnel reflection losses (around 12% for bare lithium niobate). In reality, this figure would be more complicated as the crystal would act as a low-finesse cavity for the idler.

Due to this uncertainty, in Fig. S8c/d we perform sweeps over intrinsic loss to investigate the effects on the phenomena we describe. We observe relative robustness to even relatively high levels of intrinsic loss. Plotted is the enhancement factor for the example application of "remote sensing spectroscopy" in Sec. C C.1 (shown for each idler comb line used as a probe), alongside nullifier metrics for two example graph states investigated in the main text (see also Fig. S8). As per the heuristic argument above, the outcoupling loss is quite high (here 50%) and the pump powers are in the kW range, though this is still well within the regime of quasi-CW nanosecond sources.

H.5. Pump and dissipation engineering

The main text explores approaches pump and dissipation engineering as plausible ways to shape the covariance matrix of nonlinear multimode systems. Pump engineering is well established: in synchronously pumped $\chi^{(2)}$ systems, the pump spectrum sets the multimode coupling matrix [S22], and optimizing pump spectral amplitude and phase has been shown to improve multimode squeezing and cluster state nullifiers under realistic pulse-shaper constraints [S23]. Experimentally, spectral pulse shaping is already standard in quantum frequency-comb work; it is used to probe and change the relevant modal basis in covariance-matrix measurements [S24].

Frequency-dependent cavity coupling, detuning, and loss/dispersion engineering are established control knobs that alter the multimode coupling landscape and therefore the attainable covariance structure. For example, it has been shown that intracavity dispersion changes the effective supermode detunings and induces linear coupling between supermodes, which in turn changes squeezing and entanglement [S25]. More broadly, dispersion and walkoff are explicit design knobs in synchronously pumped OPO theory [S26] and dispersive resonant cavities can be optimized for

nonlinear wave mixing across multiple frequencies [S27].

H.6. Comb stability

The role of comb stability depends on the implementation under discussion. In the main discrete-mode cavity analysis, we do not assume pumping by a free-running broadband mode-locked comb. Instead, we consider a narrowband quasi-CW pump that is electro-optically modulated to generate a small set of sidebands (note the small J simulated), and this EO-generated pump comb is matched to the cavity free spectral range. In that setting, the relevant experimental requirement is primarily stable locking of the pump carrier to the cavity together with synchronization of the EO modulation frequency to the cavity FSR, rather than full stabilization of an independent frequency comb. Under those conditions, the relative frequencies of the driven cavity modes are set by the modulation source and the cavity resonance structure, so the stability requirement is mainly that residual detuning remain small compared with the cavity linewidth over the measurement time. We therefore expect this architecture to be substantially less demanding than pumping with a fully broadband free-running comb. Full stabilization would still be beneficial experimentally, since it would reduce technical frequency and phase noise and improve the visibility and robustness of the measured correlations, but it is not essential to the basic discrete-mode mechanism analyzed in the main text.

For the ultrafast section, our simulations describe single-pulse nonlinear dynamics rather than a stabilized cavity frequency comb, so individual comb-line coherence is less directly applicable there. Such coherence would become more relevant if one were to realize the same physics using a mode-locked laser or soliton frequency comb coupled to a resonator, in which case full comb stabilization [S28] would indeed be advantageous.

H.7. Other materials

If adjusting the spectral range of operation is viable, other crystals may be considered:

1. **PPKTP/KTP family:**

   (a) **Properties:** Strongest non-LN candidate for multi-process phase matching. Concur-

rent second-order interactions in one poled KTP crystal have been demonstrated, and quasiperiodic KTP solved a three-process multi-phase-matching problem experimentally [S29, S30]. Fan-out PPRKTP also gives tunable QPM engineering [S31]. Higher damage threshold than PPLN.

   (b) **Necessary changes to the proposed system:** Transparency range is around 0.35-4.5 micron [S32], so the UV end should be relaxed. Mid-IR/terahertz absorption can be reduced substantially in LN and KTP with cooling [S33].

2. **OP-GaP/GaAs:**

   (a) **Properties:** Attractive for QPM mid-IR DFG/OPO: broad IR transparency, decent nonlinearity, and multi-grating/fan-out devices with OPO tuning from different grating regions have been demonstrated [S34].

   (b) **Necessary changes to the proposed system:** Short-wave cutoff is around 0.8 micron, so visible/UV combs are not supported.

I. COMPARISON TO PRIOR WORKS

In Table S2, we provide a comparison of the architecture proposed in this work to existing quantum and classical comb systems, including quantum dual comb and microcomb systems. In our system, a shared idler comb mediates cascaded three-wave mixing to generate mutual quantum correlations between many distinct, highly nondegenerate frequency combs, producing a structured, two-dimensional synthetic lattice of bright, individually-addressable entangled bands, rather than a single squeezed comb or a measurement protocol built from independent combs.

Recent years have seen the emergence of enormously broad frequency combs, including 3 octave combs spanning from UV to mid-IR [S9, S35]. In our proposed system, extreme spectral separation alone is not unique: a sufficiently broadband parametric or Kerr comb may also contain teeth at widely separated frequencies. The distinction in this work is therefore not bandwidth per se, but the hierarchical coupling topology. In a conventional broadband comb, two remote modes are embedded in a single global supermode/correlation structure [S24, S36, S37]. In the proposed "comb-of-comb" system, by contrast, distinct subcomb families are coupled through a shared idler comb and cascaded $\chi^{(2)}$ processes, producing explicit inter-band entangling links

whose strengths can be engineered through the adjacent conversion channels, loss profile, and QPM design [S3, S7]. This makes the resource qualitatively different from selecting two distant teeth of one comb: it realizes a structured synthetic lattice of spectral families, naturally suited to cases where one band probes a sample and another band provides low-noise readout. This also goes beyond our earlier single-family cascaded cavity model [S38], where the correlations lived within one resonance family rather than between multiple subcomb families.

From another perspective, in a single broadband comb, a UV tooth and a mid-IR tooth are correlated only if a single phase-matched process directly couples them (which is difficult across 3 octaves); in the proposed architecture, these teeth are connected through a cascade of phase-matched steps mediated by the shared idler, so correlations exist between bands that have no direct nonlinear coupling at all. The fact that "correlations occur between modes that never directly interact" is highlighted in the main text (Sec. III) and is a feature that to our knowledge has no parallel in single comb systems.

**Prior work class**	**Refs.**	**Main structure**	**Main quantum/functional result**	**What the present work adds**
SPOPO single comb	[S24, S36, S37, S39]	One comb, often analyzed in spectral bands or supermodes	Multimode squeezing and multipartite entanglement within a single comb; below-threshold supermodes	Explicit coupling between multiple distinct nondegenerate combs, not only within one comb
Quantum microcomb	[S40–S44]	One resonator family	Frquency-bin clusters	Multispectral clusters, distinct carrier bands
$\chi^{(2)}$ / microresonator multicolor comb generation	[S8–S10, S45, S46]	Broad classical comb generation, up/down conversion, or multicolor output	Coherent spectral translation and wide coverage	Quantum-correlation analysis for a shared-idler multi-comb network
Dual-comb spectroscopy	[S47–S50]	Two near-degenerate combs used for multi-heterodyne readout; squeezing or entanglement carried within a single comb (or independently across the two combs/between a comb and a local oscillator)	Improved spectroscopy readout, including entanglement-enhanced detection	Source-side generation of *mutual* inter-comb correlations/entanglement across many highly nondegenerate bands
Authors' previous cascaded process work	[S38]	One multimode cavity family coupled through a shared idler/bath mode (i.e. single comb)	Tunable single-mode and multimode squeezing; long-range correlations within one comb family	Generalization to several subcombs coupled by a common idler comb, enabling remote inter-comb links over much larger frequency gaps; 2 frequency dimensions

TABLE S2: **Comparison of the multi-comb system to existing quantum and classical comb systems.**

[S1] Van Loock, P. & Furusawa, A. Detecting genuine multipartite continuous-variable entanglement. *arXiv preprint quant-ph/0212052* (2002).

[S2] Gale, G., Cavallari, M. & Hache, F. Femtosecond visible optical parametric oscillator. *Journal of the Optical Society of America B* **15**, 702–714 (1998).

[S3] Chou, M.-H., Parameswaran, K., Fejer, M. M. & Brener, I. Multiple-channel wavelength conversion by use of engineered quasi-phase-matching structures in linbo3 waveguides. *Optics letters* **24**, 1157–1159 (1999).

[S4] Asobe, M., Tadanaga, O., Miyazawa, H., Nishida, Y. & Suzuki, H. Multiple quasi-phase-matched linbo3 wavelength converter with a continuously phase-modulated domain structure. *Optics letters* **28**, 558–560 (2003).

[S5] Schreiber, G. *et al.* Efficient cascaded difference frequency conversion in periodically poled ti: Linbo3 waveguides using pulsed and cw pumping. *Applied Physics B* **73**, 501–504 (2001).

[S6] Gorelik, P. V., Wong, F. N., Kolker, D. & Zondy, J.-J. Cascaded optical parametric oscillation with a dual-grating periodically poled lithium niobate crystal. *Optics letters* **31**, 2039–2041 (2006).

[S7] Tomita, I. *et al.* Properties of power dependence on low-crosstalk waveband conversion with an apodized multiperiod-qpm linbo3 device. *Optics Express* **22**, 15232–15244 (2014).

[S8] Roy, A. *et al.* Visible-to-mid-ir tunable frequency comb in nanophotonics. *Nature Communications* **14**, 6549 (2023).

[S9] Wu, T.-H. *et al.* Visible-to-ultraviolet frequency comb generation in lithium niobate nanophotonic waveguides. *Nature Photonics* **18**, 218–223 (2024).

[S10] Sekine, R. *et al.* Multi-octave frequency comb from an ultra-low-threshold nanophotonic parametric oscillator. *Nature Photonics* **19**, 1189–1195 (2025).

[S11] Wang, C. *et al.* Ultrahigh-efficiency wavelength conversion in nanophotonic periodically poled lithium niobate waveguides. *Optica* **5**, 1438–1441 (2018).

[S12] Jankowski, M. *et al.* Ultrabroadband nonlinear optics in nanophotonic periodically poled lithium niobate waveguides. *Optica* **7**, 40–46 (2020).

[S13] Mishra, J. *et al.* Ultra-broadband mid-infrared generation in dispersion-engineered thin-film lithium niobate. *Optics Express* **30**, 32752–32760 (2022).

[S14] Liu, Y. *et al.* Optical terahertz sources based on difference frequency generation in nonlinear crystals. *Crystals* **12**, 936 (2022).

[S15] Olgun, H. T. *et al.* Highly efficient generation of narrowband terahertz radiation driven by a two-spectral-line laser in ppln. *Optics letters* **47**, 2374–2377 (2022).

[S16] Burgess, I. B. *et al.* Difference-frequency generation with quantum-limited efficiency in triply-

resonant nonlinear cavities. *Optics express* **17**, 9241–9251 (2009).

[S17] Boyd, R. W., Gaeta, A. L. & Giese, E. Nonlinear optics. In *Springer Handbook of Atomic, Molecular, and Optical Physics*, 1097–1110 (Springer, 2008).

[S18] Shibuya, T. *et al.* Terahertz-wave generation using a 4-dimethylamino-n-methyl-4-stilbazolium tosylate crystal under intra-cavity conditions. *Applied physics express* **1**, 042002 (2008).

[S19] Krueger, A. Multiphoton microscopy: Turnkey femtosecond lasers fuel growth of multiphoton imaging. *Laser Focus World* **46**, 39–43 (2010).

[S20] Leidinger, M. *et al.* Comparative study on three highly sensitive absorption measurement techniques characterizing lithium niobate over its entire transparent spectral range. *Optics express* **23**, 21690–21705 (2015).

[S21] Fabre, C., Giacobino, E., Heidmann, A. & Reynaud, S. Noise characteristics of a non-degenerate optical parametric oscillator-application to quantum noise reduction. *Journal de Physique* **50**, 1209–1225 (1989).

[S22] Patera, G., Treps, N., Fabre, C. & De Valcarcel, G. J. Quantum theory of synchronously pumped type i optical parametric oscillators: characterization of the squeezed supermodes. *The European Physical Journal D* **56**, 123–140 (2010).

[S23] Arzani, F., Fabre, C. & Treps, N. Versatile engineering of multimode squeezed states by optimizing the pump spectral profile in spontaneous parametric down-conversion. *Physical Review A* **97**, 033808 (2018).

[S24] Medeiros de Araújo, R. *et al.* Full characterization of a highly multimode entangled state embedded in an optical frequency comb using pulse shaping. *Physical Review A* **89**, 053828 (2014).

[S25] Averchenko, V., Malyshev, D. & Tikhonov, K. Effect of group-velocity dispersion on the generation of multimode pulsed squeezed light in a synchronously pumped optical parametric oscillator. *New Journal of Physics* **26**, 123017 (2024).

[S26] Hamerly, R. *et al.* Reduced models and design principles for half-harmonic generation in synchronously pumped optical parametric oscillators. *Physical Review A* **94**, 063809 (2016).

[S27] Klemens, G., Chen, C.-H. & Fainman, Y. Design of optimized dispersive resonant cavities for nonlinear wave mixing. *Optics Express* **13**, 9388–9397 (2005).

[S28] Pan, T.-Y. *et al.* Boosting silica micro-rod q factor to 8.28$\times$ 109 for fully stabilizing a soliton microcomb. *Nature Communications* **16**, 8878 (2025).

[S29] Pysher, M., Bahabad, A., Peng, P., Arie, A. & Pfister, O. Quasi-phase-matched concurrent nonlinear-

ities in periodically poled k ti opo 4 for quantum computing over the optical frequency comb. *Optics letters* **35**, 565–567 (2010).

[S30] Bahabad, A., Voloch, N., Arie, A. & Lifshitz, R. Experimental confirmation of the general solution to the multiple-phase-matching problem. *Journal of the Optical Society of America B* **24**, 1916–1921 (2007).

[S31] Zukauskas, A., Pasiskevicius, V., Laurell, F. & Canalias, C. Fan-out periodically poled structures in rb-doped ktiopo4 for continuously tunable qpm devices. In *Nonlinear Frequency Generation and Conversion: Materials and Devices XVIII*, vol. 10902, 83–89 (SPIE, 2019).

[S32] Mamrashev, A. *et al.* Optical properties of ktp crystals and their potential for terahertz generation. *Crystals* **8**, 310 (2018).

[S33] Matlis, N. *et al.* Scaling narrowband thz generation to large apertures in linbo3 and ktp. *Optics Express* **32**, 33875–33893 (2024).

[S34] Schunemann, P. G. *et al.* Continuous wavelength tuning from 3.9–12 μm from an optical parametric oscillator based on orientation-patterned gap grown on gaas. *Optical Materials Express* **11**, 654–663 (2021).

[S35] Hutter, S. R., Sterk, P., Haller, S., Cimander, M. & Leitenstorfer, A. 1 phz of coherent bandwidth from a compact fiber device. *Laser & Photonics Reviews* **19**, e00355 (2025).

[S36] Roslund, J., De Araujo, R. M., Jiang, S., Fabre, C. & Treps, N. Wavelength-multiplexed quantum networks with ultrafast frequency combs. *Nature Photonics* **8**, 109–112 (2014).

[S37] Pfister, O. Continuous-variable quantum computing in the quantum optical frequency comb. *Journal of Physics B: Atomic, Molecular and Optical Physics* **53**, 012001 (2020).

[S38] Pontula, S., Salamin, Y., Roques-Carmes, C. & Soljačić, M. Shaping quantum noise through cascaded nonlinear processes in a dissipation-engineered multimode cavity. *PRX Quantum* **5**, 040345 (2024).

[S39] Chen, M., Menicucci, N. C. & Pfister, O. Experimental realization of multipartite entanglement of 60 modes of a quantum optical frequency comb. *Physical review letters* **112**, 120505 (2014).

[S40] Jia, X. *et al.* Continuous-variable multipartite entanglement in an integrated microcomb. *Nature* 1–8 (2025).

[S41] Wang, Z. *et al.* Large-scale cluster quantum microcombs. *Light: Science & Applications* **14**, 164 (2025).

[S42] Yang, Z. *et al.* A squeezed quantum microcomb on a chip. *Nature Communications* **12**, 4781 (2021).

[S43] Jahanbozorgi, M. *et al.* Generation of squeezed quantum microcomb in an integrated silicon nitride microresonator. In *Laser Science*, JW4A–53 (Optica Publishing Group, 2023).

[S44] Shen, Y. *et al.* Highly squeezed nanophotonic quantum microcombs with broadband frequency tunability. *arXiv preprint arXiv:2505.03734* (2025).

[S45] Herr, S. J. *et al.* Frequency comb up-and down-conversion in synchronously driven χ (2) optical microresonators. *Optics letters* **43**, 5745–5748 (2018).

[S46] Szabados, J. *et al.* Frequency comb generation via cascaded second-order nonlinearities in microresonators. *Physical review letters* **124**, 203902 (2020).

[S47] Shi, H. *et al.* Entanglement-enhanced dual-comb spectroscopy. *npj Quantum Information* **9**, 91 (2023).

[S48] Herman, D. I. *et al.* Squeezed dual-comb spectroscopy. *Science* **387**, 653–658 (2025).

[S49] Hariri, A. *et al.* Entangled dual-comb spectroscopy. *Physical Review X* **15**, 041009 (2025).

[S50] Wan, Z., Chen, Y., Zhang, X., Yan, M. & Zeng, H. Quantum correlation-enhanced dual-comb spectroscopy. *Light: Science & Applications* **14**, 257 (2025).
